# Disentangling the Impacts of Speciation, Sympatry and the Island Effect on the Morphology of Seven *Hynobius* sp. Salamanders

**DOI:** 10.3390/ani11010187

**Published:** 2021-01-14

**Authors:** Amaël Borzée, Mi-Sook Min

**Affiliations:** 1 Laboratory of Animal Behaviour and Conservation, College of Biology and the Environment, Nanjing Forestry University, Nanjing 210037, Jiangsu, China; 2 Research Institute for Veterinary Science, College of Veterinary Medicine, Seoul National University, Seoul 08826, Korea

**Keywords:** morphology, Hynobiid, Korean peninsula, island effect, patry, salamander

## Abstract

**Simple Summary:**

Morphological changes are common in populations of animals in response to environmental and evolutionary forces. This is the case for salamanders, which can adapt to most environments on earth. On the Korean Peninsula, *Hynobius* salamanders are widespread, with several species overlapping in distribution. In addition, while there are seven segregated clades based on mitochondrial DNA, only four of them have been described as segregated species and the three others are candidate species for which the species level of divergence has not been tested yet. Here we measured 329 individuals from all seven clades, in areas of range overlap or not, on islands and on the mainland (Graphical Abstract A), and tested for the species status of the three candidate species. Individuals on the mainland had a generally broader morphology than those on the islands (Graphical Abstract B), and individuals in the range overlap differed from the individuals from the same species that were not found in presence of another clade (Graphical Abstract C). Despite a significant impact of the island effect and the sympatric areas, all seven clades have significantly different morphologies, and we describe *Hynobius notialis* sp. nov., *Hynobius geojeensis* sp. nov. and *Hynobius perplicatus* sp. nov.

**Abstract:**

Closely related individuals from different areas can see their morphologies change based on differences between clades, but also ecological variables such as the island effect or sympatry. This is the case of salamanders, which have adapted to a broad range of ecological niches, ranging from underground dwellers in xeric landscape to tropical arboreal habitats. On the Korean Peninsula, salamanders from the *Hynobius* clade are widespread on the mainland and islands, with several partially sympatric clades and candidate species. Currently, seven lineages have been identified based on mtDNA, four of them matching named species and three others for which the species status remains untested. While the morphology of Korean *Hynobius* is known to be variable between genetically segregated clades, we hypothesise that (1) the candidate species are morphologically different, and that (2) the island effect and (3) the sympatric status have significant impacts on the morphology of individuals within the genus. Here we measured 329 *Hynobius* salamanders from all seven clades, in areas of sympatry and allopatry, and on islands and on the mainland (Graphical Abstract A). We determined that the island effect had a significant impact on the morphology of the genus, with mainland individuals generally displaying a broader range of morphology than islandic individuals (Graphical Abstract B). We also determined that sympatry had an impact on morphology, with the sizes of individuals from clades in sympatric areas diverging from each other (Graphical Abstract C). Finally, we demonstrated that all seven clades have significantly different morphologies, and we described the three candidate species that had already been isolated based on mtDNA and microsatellite data: *Hynobius notialis* sp. nov., *Hynobius geojeensis* sp. nov. and *Hynobius perplicatus* sp. nov. We conclude that looking at morphology alone would be misleading about the true diversity of *Hynobius* species, and species in general, because of the island and patry effects.

## 1. Introduction

Numerous variables can cause shifts and divergences in species’ phenotypes. Among these differences, some morphological variations result from adaptation to specific habitats and behaviours [1,2,3,4]; others are the results of indirect pressures, such as competition and niche segregation [5,6,7], especially in areas of sympatry [8,9]. Some other morphological variations are the results of non-directional drift, as illustrated in some cases of the island effect [10,11,12]. Examples of morphological shift as a result to habitat specialisation include the crab *Cyrtograpsus angulatus* in Patagonia, Argentina: the shape of the carapace of individuals on rocky shores is more slender and lengthened than that of individuals living in salt marshes [13]. Another example is that of the Olm *Proteus anguinus*, living in caves and having evolved relatively elongated limbs, advanced inner ear receptors and a generally increased lateral line system to adapt to the habitat [14,15,16]. Morphological adaptations to decrease competition are also widespread, as seen, for instance, in the South American marsupials *Didelphis altimetric* and *D. aurita*, for which significantly different skull morphometrics enable the exploitation of marginally different feeding niches. In addition, the morphological differences are reinforced in *D. aurita* in sympatric areas [17]. The same pattern is seen in amphibians, and especially for larvae in relation to habitat [18,19,20,21,22,23]. However, adaptation can happen at all life stages, and eggs size, for instance, represents a local adaptation from lentic to lotic habitats and to escape predation within populations of the small-mouthed salamander (*Ambystoma texanum*; [24]). Similarly, *Discoglossus pictus* introduced into Europe show a gradient of morphological variations covariating with habitat selection [23]. Finally, drift because of the island effect is illustrated by species on Jeju Island in the Republic of Korea, appearing darker in relation to the predation pressure (*Bombina orientalis*; [25]) or for yet undetermined reasons (*Gloydius ussuriensis* [26]), or larger for yet unknown reasons (*Dryophytes japonicus* [27,28]).

Salamanders have also undergone a massive divergence in ecology and morphology [29,30], with specific adaptations to corresponding habitats [31] highlighting a greatly plastic morphology [30,32,33]. For instance, salamanders can be found inhabiting a diversity of habitats, ranging from terrestrial niches in xeric habitats, such as *Salamandra infraimmaculata* in Israel [34], all the way to arboreal behaviours in tropical forests such as *Bolitoglossa equatoriana* in Ecuador [35]. The impact of the island effect is also visible in salamanders, and *Pachyhynobius shangchengensis’* isolation to the sky islands of East China, for instance, resulted in strongly divergent morphology [36,37,38]. Specifically related to this study, several North American *Desmognathus* salamanders species can be found in sympatry, with ecologically partitioned guilds within streams, ranging from large bodied species that are more aquatic to smaller bodied species that are more terrestrial [39]. In opposition, *Plethodon* salamanders have non-adaptively radiated, resulting in segregated species with similar morphologies, partially overlapping ranges and gene exchange at the extremities of these ranges [40].

The Korean Peninsula is rich in hynobiid salamanders, inhabited by both *Onychodactylus* and *Hynobius* genera. The divergence of both genera within the Korean Peninsula resulted in independent clades, with three described and one candidate *Onychodactylus* species [41], and four described and three candidate *Hynobius* species [42]. The *Hynobius* genus is the most speciose genus of the Hynobiidae family [43,44] and it originated in the early Cenozoic [45]. While some variability is known between the currently described *Hynobius* clades [46,47,48,49,50] and Korean clades [42,51,52], the origins of such variation are not yet determined. Here, we test whether (1) the morphological variation encountered in Korean *Hynobius* relates to genetic divergence and solely groups within clades; whether it is also related to (2) the island effect and (3) the patry of populations; and whether morphological variation is linked to mtDNA clades but mitigated by the island and patry effects. In addition, our results enabled the descriptions of the three candidate species, following calls for species descriptions from numerous publications [42,51,53,54,55].

## 2. Materials and Methods

**Figure 1:**
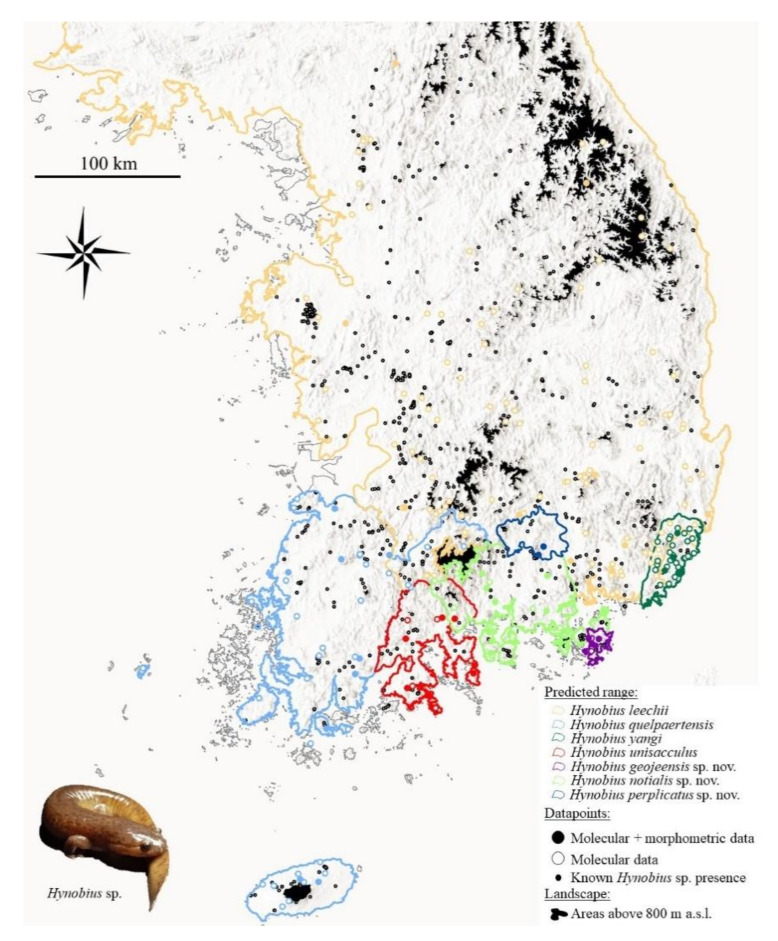
Sampling sites and predicted ranges. Map representative of sites with molecular and morphometric data, molecular data only and presence of *Hynobius* sp. The predicted ranges are drawn to be continuous until landscape barriers preventing the dispersion of the genus (>800 m a.s.l. and sea water). As there was more than one sample per site, the number of points on the map does not match with the number of individuals sampled. Map created in ArcMap 10.6 (desktop.arcgis.com; ESRI, Redlands, CA, USA).

A total of 329 *Hynobius* individuals were preserved as vouchers between 2008 and 2013; originating from five sampling sites for *H. yangi* (*n* = 23), three sampling sites for *H. geojeensis* sp. nov. (HC3 in previous publications [42]; *n* = 29), 16 sampling sites for *H. notialis* sp. nov. (HC1 in previous publications [42]; *n* = 93), seven sampling sites for *H. unisacculus* (*n* = 52), ten sampling sites for *H. quelpaertensis* (*n* = 43), 11 sampling sites for *H. leechii* (*n* = 46) and two sampling sites for *H. perplicatus* sp. nov. (HC4 in previous publications [42]; *n* = 43; Figure 1).

As all vouchers were similarly preserved in 70% alcohol since sampling, shrinking because of dehydration was not considered a problem [56]. However, the data collected here cannot be compared with living animals, or collections relying on other fixatives. Tissue (leg or tail muscle) was extracted from each individual for species identification with molecular tools and all sequences are already available in the literature [42,52,53,54,55,57].

### 2.1. Data Collection Adults

Morphometric data were collected with digital callipers (1108–150, Insize; Suzhou, China) to the nearest 0.1 mm, and follow the recommendations in methodology and characteristics measured [42,48]. Each individual was measured three times by the same observer to ensure accuracy, but tail length could not be measured for all individuals as some of the tails were previously removed for DNA extraction. The morphometric measurements collected were: SVL—snout-vent-length; TL—tail length; GA—gleno–acetabular distance (minimum distance between axilla and groin measured on a straightened body); CW—chest width (minimum distance between left and right axillae); FLL—forelimb length (length of the straightened forelimb measured from axilla to tip of the longest finger of forelimb); HLL—hindlimb length (length of the straightened hindlimb from groin to tip of the longest toe of hindlimb); HL—head length; HW—head width; EL—eye length (minimum distance from the anterior corner of the eye to the posterior corner of the eye); IN—inter-nostril distance; ON—orbitonarial distance (minimum distance between external nares and the anterior corner of the eye on the same side of the head); IO—interorbital distance (minimum distance between the upper eyelids); OR—orbitorostral distance, snout length (measured as minimum distance from tip of snout to the anterior corner of the eye); IC—intercanthal distance (measured as minimum distance between anterior corners of the eyes).

For the subsequent holotype description, in line with the last *Hynobius* species description from the Korean Peninsula [42], we recorded the following characteristics (following [48]): 1-FL—first finger length; 2-FL—second finger length; 3-FL—third finger length; 4-FL—fourth finger length; 1-TL—first toe length; 2-TL—second toe length; 3-TL—third toe length; 4-TL—fourth toe length; 5-TL—fifth toe length; MTH—tail height in the middle; MTW—tail width in the middle; MAXTH—maximal tail height. Meristic characteristics recorded were counted for the left body side of each individual and included: CGN—costal grooves number (number of costal grooves between the forelimbs and hindlimbs, excluding axillary and inguinal grooves, following [58]) and TGN—tail groove number (number of clearly discernible grooves on lateral sides of tail). We were not able to determine the sex of all specimens used in this study, and the variable sex was therefore discarded for all analyses. Including this variable would have likely resulted in an increase in variation between the groups analysed, but not the opposite, and not including sex here only highlights the robustness of the dataset.

### 2.2. Range Reconstruction

To assess the patry status of each individual—here defined as sympatric or allopatric—independently of the presence of samples from the location belonging to two clades, we reconstructed the range of each species. To do so, we used all datapoints with molecular identification available from the literature [42,52,53,54,55,57]. However, some datapoints were not precise enough to be used for range reconstruction, and we only used datapoint with at least 2 km accuracy, resulting in a sample size such as: *H. leechii* = 107; *H. quelpaertensis* = 31; *H. unisacculus* = 5; *H. yangi* = 29; *H. notialis* sp. nov. = 10; *H. geojeensis* sp. nov. = 2 and *H. perplicatus* sp. nov. = 2. We did not use alternative data, such as data originating from citizen science, as it has been noted to be unreliable for newly described taxa for our focal species [59]. However, we downloaded datapoints from GBIF.org to populate range maps with presence points (doi:10.15468/dl.rxx5kn; Figure 1).

The ranges were defined such as the extent of land on which a population assigned to a species was found, until encountering a barrier to the dispersion of the species. Dispersion barriers for the species were defined as any saline environment, river larger than 20 m wide, and areas above 800 m a.s.l. Saline environments were selected as a barrier to dispersal because no hynobiid species is known to be salt tolerant. Rivers with a width > 20 m were selected arbitrarily, 20 m being a very conservative estimate, as salamanders are known to be poor disperser, with most individuals unlikely to move more than 10 m [60], and seldomly moving more than 20 to 30 m away from their habitat [61]. More specifically, *Hynobius* sp. are unlikely to disperse more than 100 m [62]. However, we were not able to acquire any database that could be restricted for rivers wider than 20 m, and to follow the protocol we measured the width of each river being a potential barrier to dispersion every 100 m on Google Earth (Google; Mountain View, CA, USA). We selected a maximum elevation of 800 m to the distribution of the species as the genus is not found above 776 m a.s.l. ([63]; and data available from the GBIF dataset). Urban areas are also permeable to the dispersion of *Hynobius* sp., and may impact connectivity, but we estimate that they are too recent to have impacted the range of the species. For the range reconstruction, we did not consider the absence of datapoint as a substitute for the absence of *Hynobius* sp.—instead, it was only as a demonstration of limited sampling—and it is possible that changes in the river system because of geological events and human activities may have blurred the exact boundaries of the species range. Based on the ranges reconstructed here, each datapoint was assigned to either the islandic (here also including the Goheung and Tongyeong peninsulas (Graphical Abstract) due to the narrow dispersion routes) or continental group. We also assigned each individual to observed sympatry or allopatry, based on syntopy from the molecular data available, and to putative sympatry or allopatry based on range reconstruction. However, a single individual belonging to the *H. yangi* clade (mms8151) was assigned to a different patry status when assessed through putative sympatry or allopatry based on the dataset, and we did not pursue the analysis based on putative sympatric data.

### 2.3. Data Collection for Eggs and Larvae

To ensure convergence of development traits for all *Hynobius* clades from the Korean Peninsula, we compared the tadpoles of all clades. We took pictures of at least three samples for each focal development stage, making sure the tadpoles originated from unrelated egg sacs to maintain independence of samples. We photographed tadpoles between stages 29 and 37 while the embryo was still in the egg sac; tadpoles between stages 40 and 49 when the larvae were free swimming with balancers; and tadpoles between stages 50 and 59, when the larvae were not yet using their developing hind legs [64]. Differences in development were visually assessed to determine whether eggs and larvae of all clades develop similarly.

### 2.4. Genetic Reconstruction

To illustrate the genetic relationship between the clades and being able to reconstruct ranges in the next step, we reconstructed phylogenetic trees including all seven *Hynobius* clades. The first integrated tree constructed for Korean *Hynobius* was based on 1706 bp for the concatenated mitochondrial genes (Cyt-*b* + 12S rRNA; [53]), and the resulting tree was built upon to include *H. unisacculus*, also highlighting the presence of two candidate species: HC1 and HC3 [42]. Later on, additional analyses based on COI, Cyt-*b*, D-loop and microsatellites confirmed the isolation of the two candidate species, and highlighted the presence of a third candidate species: HC4 [55]. Unfortunately, the gene fragments used for the different analyses did not overlap, and a tree with concatenated sequences could not be built. The trees redrawn for the purpose of this paper were therefore extracted from [55] with the only difference being that the same graphic space was given in the tree (i.e., sample size) for each clade.

### 2.5. Data Preparation and Analysis

As body size of small vertebrates is known to be affected by climate [65], and some of the measurements collected here were relatively old and potentially impacted by climate change, we decided to use a ratio to correct for body size in relation with individual growth, in line with the general literature on morphological analyses [10]. This decision was made in agreement with earlier publication comparing the use of ratios to correct for isometric growth between individuals of different age classes [66,67], and because not all individuals collected were from the same age and sex, and vouchers are therefore expected to display more variation than a single age class (e.g., breeding males; [68]). To create the ratio, we did not divide the data by the total body length (SVL + TL) because of missing data for TL (*n* = 36), but by SVL only.

To harness as much of the variation possible expressed by the 13 morphological measurements collected for the 329 individuals, and because 62 out of 72 measurements were correlated (Pearson correlation; Table 1), we opted for a factor reduction analysis. We selected a principal component analysis (PCA), and tested the resulting principal components (PCs) against the categories described above to answer each for the three hypotheses. Due to the large number of missing data for TL (*n* = 36), we first tested the percentage of variance expressed by each of the PCs based on a dataset with the variable TL included but all individuals with missing TL data excluded from the dataset (293 individuals; 13 variables). We then compared the variance expressed with that of the dataset from which we had excluded TL but included all individuals (329 individuals; 12 variables). The PCAs were set such that PCs were to be extracted if their eigenvalues were >1, under varimax rotation and with Kaiser normalisation. The PCA with TL resulted in a cumulative percentage of 60.09, and the dataset without TL expressed only 53.49% of the variance (Table 2). We consequently based the subsequent analyses on the dataset including TL, despite the lower number of individuals included in the analyses. The rotations converged in seven iterations and variables were selected as loading into a PC if > 0.47 (Table 3).

Once the PCs were extracted, we tested for significant differences between groups based on the three hypotheses. To test for variations between clades we used a one-way ANOVA with PCs as dependent variables and clades as independent variables. In addition, we conducted Tukey post-hoc tests to determine the presence of significant variations between the seven clades. Finally, we compared the average sizes of the variables measured to assess the possibility of identifying each clade based on specific morphological features.

To test for variations between mainland and islandic populations, we used a generalised linear model employing a linear scale response variable. The islandic binary variable was used as dependent variable; however, we scaled it with the binary sympatric variable to removed bias, and offset the covariates with the variable clade to correct for the impact of inter-clade variations on the dataset. The covariates in this analysis were the four PCs extracted from the PCA, and the analysis was run under a main effect model.

To test for variations between sympatric and allopatric populations, we used the same statistical analysis, employing a generalised linear model with a linear scale response variable. The sympatric binary variable was used as dependent variable; however, we scaled it with the variable “sympatric with” to remove bias, and we offset the covariates with the variable clade to correct for the impact of inter-clade variations on the dataset. The covariates in this analysis were the four PCs extracted from the PCA, and the analysis was run under a main effect model.

We jointly tested the assumptions for both GLMM, and found by visual inspection of scatterplots and partial regression plots that there were linear relationships between the dependent variable and each independent variable, or a relationship between the dependent variable and independent variables collectively. The data demonstrated homoscedasticity, visually inspected through the scatterplot of the regressed standardised predicted values plotted against the regressed standardised residuals. Values of skewness and kurtsosis were below standard error values and were not collinear, displaying tolerance values between 0.19 and 0.76 (>0.1 indicating non-collinearity). Variance Inflation Factor values were between 3.54 and 8.56 (<10 indicating non-collinearity; [69,70]).

We then restricted the dataset to populations in sympatry only (*H. unisacculus n* = 3; *H. notialis* sp. nov. *n* = 21; *H. geojeensis* sp. nov. *n* = 30; *H. perplicatus* sp. nov. *n* = 17) and conducted a one-way ANOVA with PCs as dependent variables and “clades in sympatry with” as the independent variable with Tukey post-hoc tests to assess for variations between clades. As *H. notialis* sp. nov. demonstrated significant intra-clade variation between sympatric and allopatric populations, and because it is the only clade overlapping with three others, we then restricted the dataset to *H. notialis* sp. nov. and sympatric species and compared the variations in size based on the other clade it was overlapping with. All biostatistical analyses were conducted in SPSS (SPSS, Inc., Chicago, IL, USA).

## 3. Results

Generally, most clades shared an area of sympatry with their neighbours, and all analyses showed significant differences for some variables, highlighting morphological differences based on clade, island effect and sympatry in *Hynobius* sp. on the southern edge of the Korean Peninsula. The PCA resulted in four PCs (Table 3), with PC1 being representative of the general body structure, PC2 being representative of the head structure, PC3 being representative of limbs and PC4 being representative of the gleno–acetabular distance. However, a variables loaded similarly in two PCs: HL loaded secondarily into PC4 (Table 3).

### 3.1. Range Reconstitution

The range reconstitution based on the criteria used resulted in the resolution of ranges for all species. There was no area that was not assigned to a clade, and thus there were no false-negative areas (type II error, i.e., an area not assigned to a species despite the known presence of the genus). Ranges were not strictly delineated for two minor exceptions: two gaps (<500 m) were left in between rivers and the limit elevation east and west of the Jiri Mountain range (see Graphical Abstract). The gaps were between *H. leechii* and *H. notialis* sp. nov., and between *H. leechii* and *H. quelpaertensis*. In these cases, the range borders were drawn following the shortest mountain crest between the two landscape features restricting the ranges of the species. The absence of a datapoint in the riverine area on the west coast, north of *H. quelpaertensis* range, matches with the intertidal area reclaimed during the past centuries and is not a false-negative. All clades were found to share an area of sympatry, with the exception of *H. quelpaertensis* and *H. unisacculus*, for which we did not detect any despite their probable existence.

### 3.2. Inter-Clade Variation

The ANOVA to test for variations between clades based on the four PCs was significant for PC1 (χ^2^ = 12.87, *F*_(286,292)_ = 17.13, *p* < 0.001), PC2 (χ^2^ = 5.20, *F*_(286,292)_ = 5.71, *p* < 0.001) and PC3 (χ^2^ = 5.01, *F*_(286,292)_ = 5.47, *p* < 0.001), but not for PC4 (χ^2^ = 1.01, *F*_(286,292)_ = 1.01, *p* = 0.418). The Tukey tests indicated a large variation in the comparison of clades two-by-two (Table 4). For instance, the pair *Hynobius unisacculus*–*H. perplicatus* sp. nov. was the pair with the highest number of significantly different PCs, while the pairs *H. leechii–H. quelpaertensis* and *H. quelpaertensis–H. yangi* were not significantly different (two-by-two) for the PCs. All species pairs with shared boundaries were significantly different for at least one PC, although non-sympatric species were not always significantly different. For instance, *H. notialis* sp. nov. did not differ significantly for any PC with *H. quelpaertensis* and *H. yangi*. Consequently, *H. leechii*, *H. quelpaertensis* and *H. yangi* had the lowest numbers of significantly different PCs from the other clades (Table 4).

**Figure 2:**
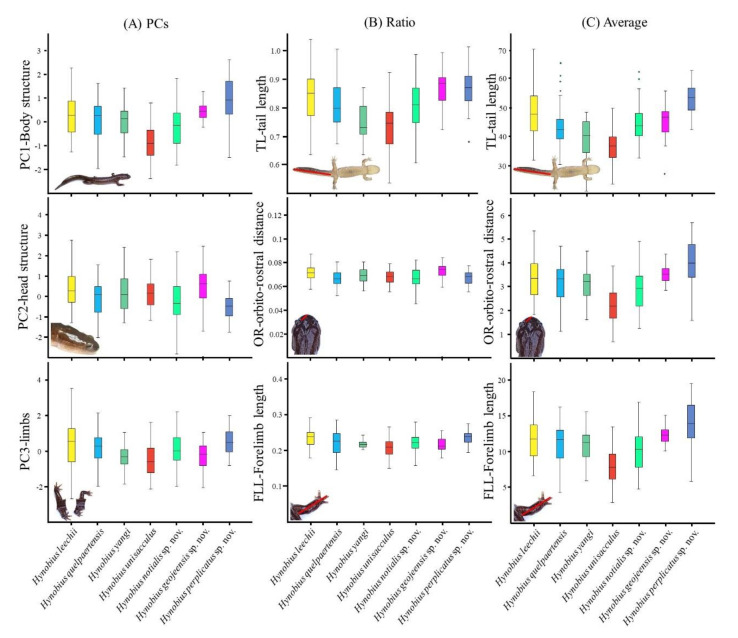
Box plots of morphological variables of importance segregated by clade. This figure includes values for PCs resulting from the PCA (**A**), ratios when morphological traits were controlled by size (**B**) and averages of real size (**C**). Only PC1, PC2 and PC3 are presented, as PC4 was not significantly different based on clade, and the variables TL, OR and FLL are used here as proxies for PC1, 2 and 3 respectively, due to their highest loading values.

The box plots computed from the three significant PCs for the seven clades (Figure 2A) highlighted the presence of subtle morphological differences among clades, and the PCs did not cluster following the focal clades. Additional box plots on the ratio (Figure 2B) and averages data (Figure 2C) obtained from each variable loading the most in terms of any of the three PCs followed the same pattern in the absence of clear distinctions for any of the clades or genetic clusters. We, however, noted that *H. perplicatus* sp. nov. and *H. leechii* were in general larger, or displayed higher values in ratios than the other clades, with *H. unisacculus* displaying the lowest values. In addition, we did not find variables that were non-overlapping for any of the clades (Table 5), despite some weakly overlapping variables when looking at all species within the range (visualised in Supplementary Materials Figure S1 and ratios in Figure S2). For species identification in sympatric area, see the sections on the impact of sympatry on morphological variables and the species descriptions.

### 3.3. Impact of the Island Effect

**Figure 3:**
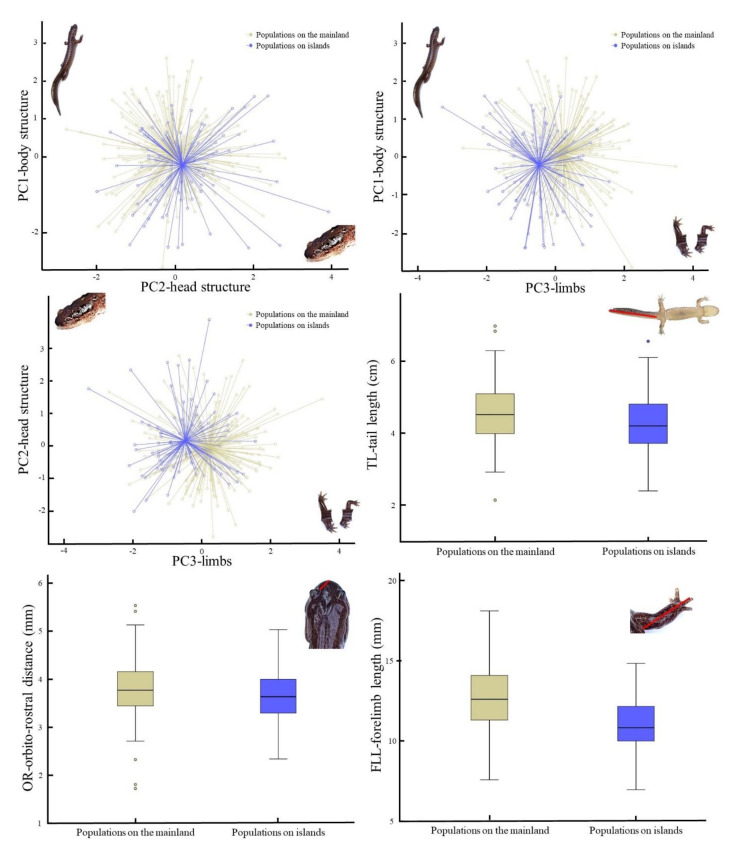
Centroids and box plots of morphological variables based on islandic distribution. This figure includes PC and real morphological values for PC1, PC2 and PC3 only, as PC4 was not significantly different based on islandic distribution. The variables TL, OR and FLL are used here as proxies for PC1, 2 and 3 respectively due to their highest loading values.

The results of the analysis to determine the impact of the island effect on the morphology of *Hynobius* spp. through the GLMM resulted in a statistically supported model (Omnibus test; *χ*^2^ = 25.33, df = 4, *p* < 0.001) when comparing the fitted model against the intercept-only model. Within the predictive variables, PC1, PC2 and PC3 were significant (Table 6). The graphs plotting the PCs against one another in relation to presence on islands or on the mainland show a clear segregation of centroids (Figure 3). Accordingly, the box plots for the variables loading the most importantly into each of the significant PCs showed a difference as well, with populations on the mainland being larger than those on islands (Figure 3; Table 5).

### 3.4. Impact of Sympatry

**Figure 4:**
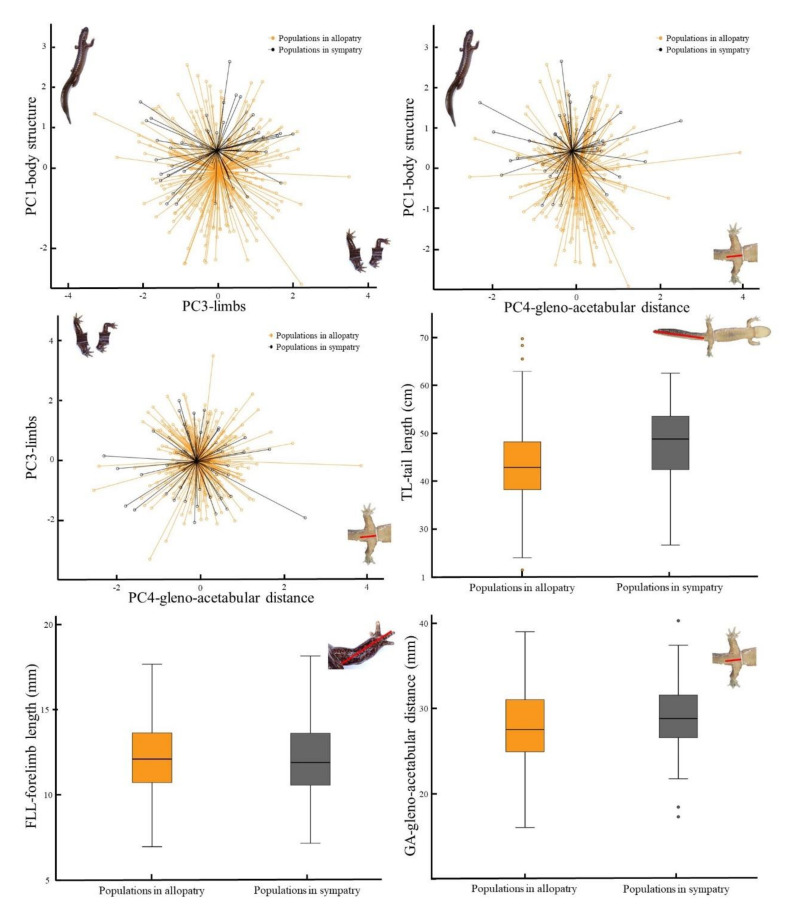
Centroids and box plots of morphological variables based on patry. This figure includes PC and real morphological values for PC1, PC3 and PC4 only, as PC2 was not significantly different based on patry. The variables TL, OR and GA are used here as proxies for PC1, 2 and 3 respectively due to their highest loading values.

The results of the analysis to determine the impact of sympatry on the morphology of *Hynobius* spp. through the GLMM resulted in a statistically supported model (Omnibus test; *χ*^2^ = 14.67, df = 4, *p =* 0.003) when comparing the fitted model against the intercept-only model. Among the predictive variables, PC1, PC3 and PC4 were significant (Table 7), with non-overlapping centroids when plotting the PCs against another (Figure 4). In terms of morphology for the variables loading the most importantly into each PC, individuals in sympatric populations showed higher values than those from populations in allopatry for tail length and gleno–acetabular region, but populations in allopatry had longer forelimb length (Figure 4; Table 5).

**Figure 5:**
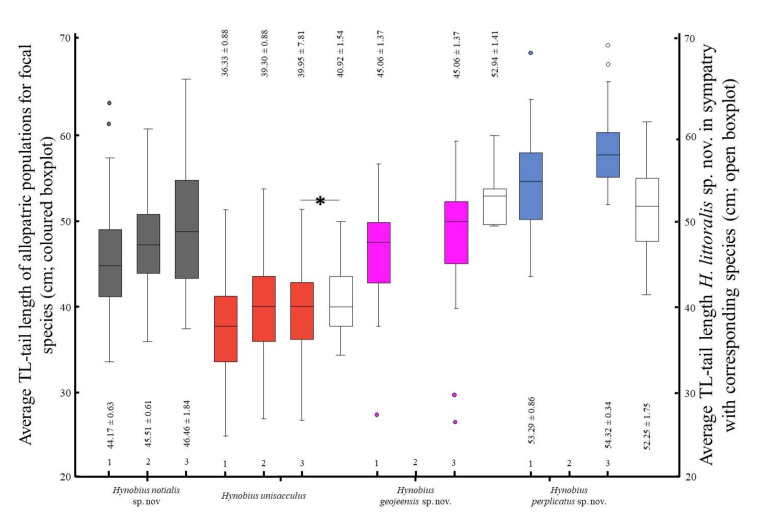
Impact of patry on the morphologies of sympatric populations. Box plots representative of the variation in tail length, as a proxy for PC1—being the only significantly different PC between clades when looking at patry independently. Here, *Hynobius notialis* sp. nov. is used as reference, as it is the only species sympatric with three other species in parts of its range. The ranges of values for the three clades overlapping with *H. notialis* sp. nov. were different when not in sympatry with *H. notialis* sp. nov. On the *x*-axis, (1) refers to all individuals of the species, (2) to allopatric populations and (3) to populations sympatric with *H. notialis* sp. nov. The asterisk indicates significant difference under the Tukey test (Table 8). Values either below or above the box plots are mean ± SD. Box plots follow the earlier species-specific colour coding.

The subsequent ANOVA on the dataset restricted to sympatric population was significant for PC1 only (χ^2^ = 1.48, *F*_(56,59)_ = 2.90, *p* = 0.043). The other PCs were not significant: PC2 (χ^2^ = 0.76, *F*_(56,59)_ = 0.96, *p* = 0.417), PC3 (χ^2^ = 1.99, *F*_(56,59)_ = 2.14, *p* = 0.104) and PC4 (χ^2^ = 0.87, *F*_(56,59)_ = 1.38, *p* = 0.256). The Tukey tests indicated a significant variation within a species pair only: *H. unisacculus*–*H. notialis* sp. nov. (Table 8). The subsequent comparison regarding the impact of sympatry on morphology using *H. notialis* sp. nov. showed that when in sympatry with another clade, the morphology of *H. notialis* sp. nov. individuals shifted towards that of the sympatric species. Namely, the range of TL, as the representative variable for PC1 was the lowest when in sympatry with *H. unisacculus* (Figure 5).

### 3.5. Eggs and Larvae

**Figure 6:**
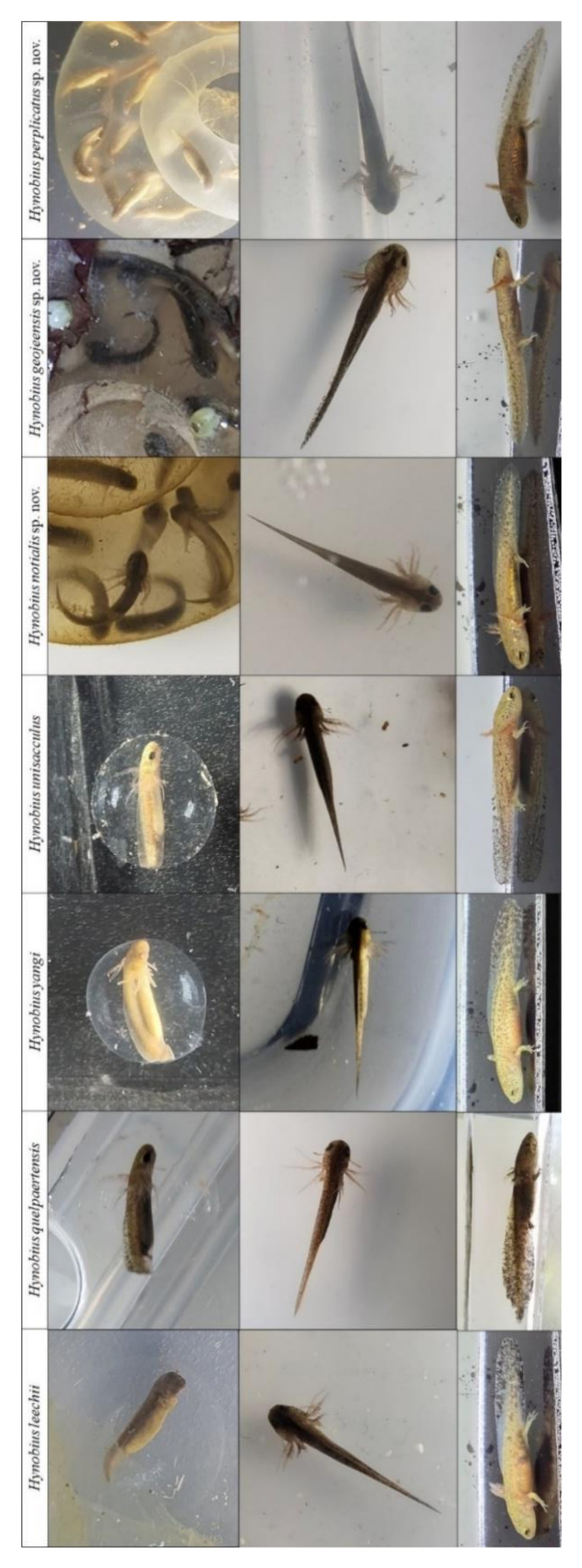
Pictures of representative eggs and larvae. We compared the general anatomy of eggs and larvae for each species of the genus on the Korean Peninsula. Eggs were photographed between stages 29 and 37, while the embryo was still in the egg sac. Larvae were then photographed between stages 40 and 49, when the larvae was free swimming with balancers, and between stages 50 and 59, when the hind legs of the larvae were developing.

The visual inspection of eggs and larvae (Figure 6) to assess whether eggs and larvae of all clades develop similarly showed no difference in the general development pattern. No differences could be found between embryos between stages 29 and 37; all larvae had balancers between stages 40 and 49; and individuals had the same number of toes and followed the same development patterns between stages 50 and 59.

### 3.6. Genetic Reconstruction

**Figure 7:**
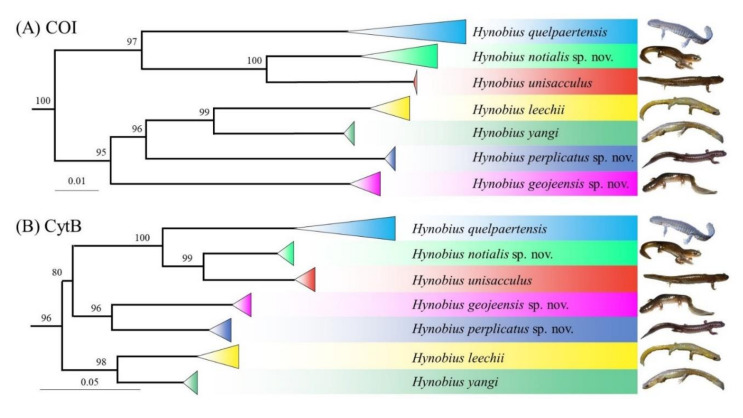
Phylogenetic tree of Hynobius sp. from the Korean peninsula. (**A**) Tree based on the COI gene fragment, modified from Min et al. (2016). (**B**) Tree based on the CytB gene fragment, modified from Suk et al. (2019).

A profound divergence has been reported between all five clades based on two mtDNA gene fragments, three nuclear gene fragments and microsatellite data [42,53,54,55]. In addition, *H. geojeensis* sp. nov. has been described as reciprocally monophyletic with *H. unisacculus*, and as a sister lineage to *H. notialis* sp. nov. with 6.8% divergence based on Cyt-*b* gene fragment and 5.6% divergence based on COI gene fragments (Figure 7). Following [55], *H. quelpaertensis*, *H. unisacculus* and *H. notialis* sp. nov. are part of a cluster, while *H. yangi* is isolated. The other clades, *H. geojeensis* sp. nov. and *H. perplicatus* sp. nov., cluster with either *H. quelpaertensis* or *H. yangi* based on the gene fragment of reference.

### 3.7. Species Description

Following the line of evidence based on molecular analyses for species-level divergence for the cryptic clades *H. notialis* sp. nov., *H. geojeensis* sp. nov. [42] and *H. perplicatus* sp. nov. [42,55], and the evidence for morphological differences between all seven clades presented in this work, we formally describe three new species. Measurements are summarised in Table 9.

#### 3.7.1. Nomenclature History

Among the currently recognised species of *Hynobius* (see [44]), *Hynobius turkestanicus* Nikolskii appears to be the only enigmatic taxon (see [71]) and it is unlikely to be a member of the *Hynobius* genus [42]. *Hynobius* species are divided into two groups relying on different habitats: one relying on lotic habitat in northern Japan and markedly different from the *Hynobius* species found on the Korean Peninsula and China, relying on lentic habitat for breeding. The three new species belong to the lentic restricted group, and morphological comparisons with the species closely related in term of genetic and geography are provided in Table 5 and Figures S1 and S2.

#### 3.7.2. *Hynobius notialis* sp. nov. Min and Borzée, 2020

Synonymy:

“*Hynobius leechii* (partim): [72]: p. 33; [73]: p. 20”.

“*Hynobius quelpaertensis* (partim): [52]: p. 1166”.

“*Hynobius leechii* Clade HC1: [53]: pp. 25–32; [54]: p. 108; [42]: pp. 475–503; [55]: pp. 165–178”.

● Holotype

**Figure 8:**
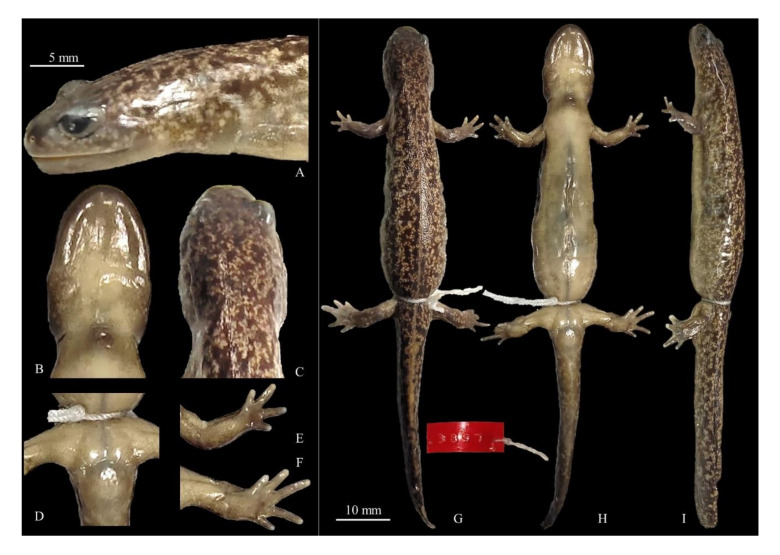
Details and holotype for *Hynobius notialis* sp. nov. Voucher CGRB15873 (field ID mms3897); adult collected on 14 March 2012 in Jangmok-myeon, Geoje-si, South Gyeongsang Province, Republic of Korea (34.986877° N, 128.682033° E). (**A**) Head in lateral view; (**B**) head in ventral view; (**C**) head in dorsal view; (**D**) ventral view of vent; (**E**) plantar view of the left hand; (**F**) plantar view of the left foot; (**G**) dorsal view; (**H**) ventral view; (**I**) lateral view. (**A**–**F**) match with the 5 mm scale bar and (**G**–**I**) match with the 10 mm scale bar. Measurements and counts in Table 9.

Voucher CGRB15873 (deposited in the Conservation Genome Resources Bank for Korean Wildlife (CGRB), College of Veterinary Medicine, Seoul National University; field ID mms3897); adult collected by Mi-Sook Min, HaeJun Baek and Dong Youn Kim on 14 March 2012 in Jangmok-myeon, Geoje-si, Gyeongsang Nam-do, Republic of Korea (34.986877° N, 128.682033° E; Figure 8). Measurements and counts in Table 9.

● Paratypes

Vouchers CGRB15870, CGRB15859, CGRB15884 (deposited in the Conservation Genome Resources Bank for Korean Wildlife (CGRB), College of Veterinary Medicine, Seoul National University; field IDs mms3894, mms3136 and mms4036 respectively). CGRB15870 adult collected by Mi-Sook Min on 14 March 2012 in Jangmok-myeon, Geoje-si, Gyeongsang Nam-do, Republic of Korea (34.986877° N, 128.682033° E). CGRB15859 adult collected by Mi-Sook Min, HaeJun Baek and Dong Yoon Kim on 5 April 2011 in Sicheon-myeon, Sancheong-gun, South Gyeongsang Province, Republic of Korea (35.27849° N, 127.840687° E). CGRB15884 adult collected by Mi-Sook Min, HaeJun Baek and Dong Youn Kim on 21 March 2012 in Jisu-myeon, Jinju-si, Gyeongsang Nam-do, Republic of Korea (35.235064° N, 128.269611° E). Measurements and counts in Table 9 illustrations in Figure S3.

● Etymology

The species was first found in Tongyeong and Namhae (Graphical Abstract), in the central costal region of the Republic of Korea. The name *H. notialis* sp. nov. comes from the southern location of the holotype, and the majority of the species’ range. The vernacular name of the species, Southern Korean salamander, reflects the scientific name of the special, as does its Korean name: 남방도롱뇽 (pronounced: Nambang Dorongnyong).

● Identity, diagnosis and distribution

To date, the species is known to range from the feet of Jiri Mountain on the north west, Gwangyang on the south west, Masan on the south east and Haman on the north east (Graphical Abstract). The species is currently known to be present on islands of Namhae, Changseong and Geoje. This species is sympatric with *H. geojeensis* sp. nov. on Geoje island, with *H. unisacculus* on Baekun Mountain north of Gwangyang and with *H. perplicatus* along the southern range of that species (Figure 1 and Graphical Abstract). Sympatry with the other Korean *Hynobius* species has so far not been demonstrated but is not impossible.

**Figure 9:**
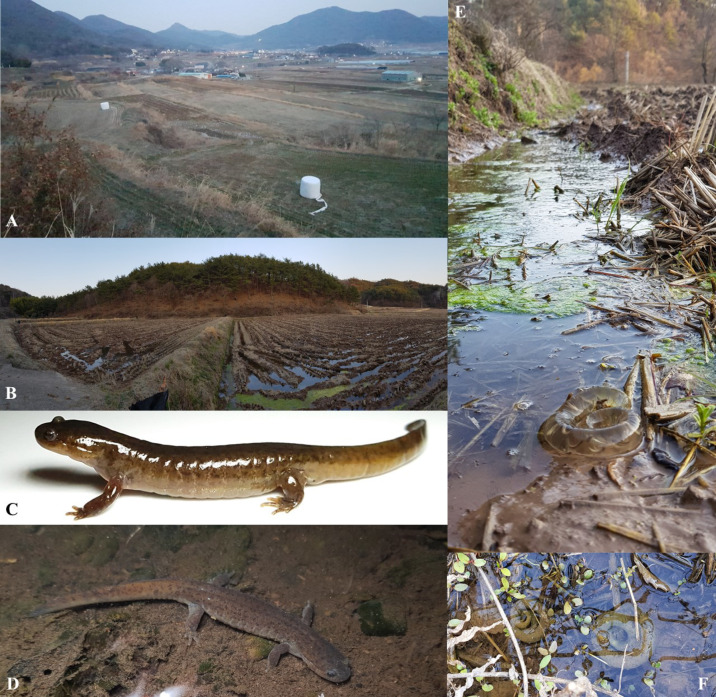
Representative habitat in life adult, and larvae and eggs for *Hynobius notialis* sp. nov. (**A**) Habitat during the breeding season (35.095278° N, 128.329167° E on 21 February 2020). (**B**) Habitat during the breeding season (35.133889° N, 128.223333° E on 18 March 2020). (**C**) Adult in situ setting from site (**B**). (**D**) Adult at site (**A**). (**E**) Eggs emerged from the water at breeding site (**B**) following drought at the site. (**F**) Sites in situ at site (**B**).

The species identification is primarily based on range, although the species is sympatric with *H. perplicatus* sp. nov., *H. geojeensis* sp. nov. and *H. unisacculus*. The species is generally larger than *H. unisacculus* and smaller than *H. perplicatus* sp. nov. (Table 5 and Table 9). However, size strongly overlaps with that of *H. geojeensis* sp. nov., despite a lower average (Figure 2; Figures S1 and S2). When in sympatry with *H. unisacculus*, the species can be identified if (in mm): SVL > 61.29, TL > 50.15, GA > 31.92, CW > 10. 72, HL > 14.00, HW > 10.83 (Table 5). For identification when in sympatry with the two other species described in this work, please refer to the matching species description. The species breeds in natural and agricultural wetlands (Figure 9).

● ZooBank registration

We hereby state that the present paper has been registered to the Official Register of Zoological Nomenclature (ZooBank) under LSID: urn:lsid:zoobank.org:pub:E0E65311-CB76-4AAE-9720-CDF1E6473E71. The new species name *Hynobius notialis* sp. nov. has been registered under LSID: urn:lsid:zoobank.org:act:82240BF1-7EFB-4DE3-A977-DCB8D4B8E841.

● Nomenclatural acts

The electronic edition of this article conforms to the requirements of the amended International Code of Zoological Nomenclature, and hence the new names contained herein are available under that code from the electronic edition of this article. This published work and the nomenclatural acts it contains have been registered in ZooBank, the online registration system for the ICZN. The ZooBank LSIDs (Life Science Identifiers) can be resolved and the associated information viewed through any standard web browser by appending the LSID to the prefix “http://zoobank.org/”. The LSID for this publication is: urn:lsid:zoobank.org:pub:E0E65311-CB76-4AAE-9720-CDF1E6473E71. The electronic edition of this work was published in a journal with an ISSN, and has been archived and is available from the following digital repositories: PubMed Central.

#### 3.7.3. *Hynobius geojeensis* sp. nov. Min and Borzée, 2020

Synonymy:

“*Hynobius leechii* (partim): [72]: p. 33; [73]: p. 20”.

“*Hynobius quelpaertensis* (partim): [52]: p. 1166”.

“*Hynobius leechii* Clade HC3: [53]: pp. 25–32; [54]: p. 108; [42]: pp. 475–503; [55]: pp. 165–178”.

● Holotype

**Figure 10:**
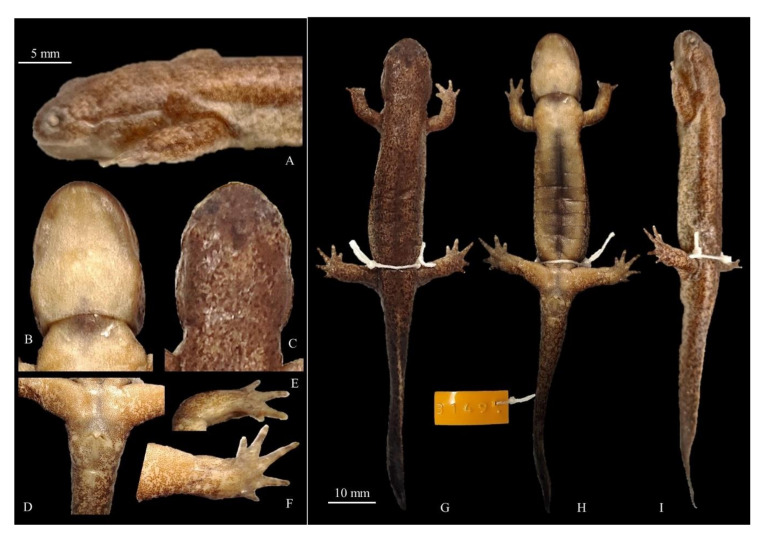
Details and holotype for *Hynobius geojeensis* sp. nov. Voucher CGRB15863 (field ID mms3149); adult collected on 6 April 2011 in Geoje-myeon, Geoje-si, South Gyeongsang Province, Republic of Korea (34.850992° N, 128.590398° E). (**A**) Head in lateral view; (**B**) head in ventral view; (**C**) head in dorsal view; (**D**) ventral view of vent; (**E**) volar view of the left hand; (**F**) volar view of the left foot; (**G**) dorsal view; (**H**) ventral view; (**I**) lateral view. (**A**–**F**) match with the 5 mm scale bar and (**G**–**I**). Measurements and counts in Table 9.

Voucher CGRB15863 (deposited in the Conservation Genome Resources Bank for Korean Wildlife (CGRB), College of Veterinary Medicine, Seoul National University; field ID mms3149); adult collected by Mi-Sook Min, HaeJun Baek and Dong Youn Kim on 6 April 2011 in Geoje-myeon, Geoje-si, Gyeongsang Nam-do, Republic of Korea (34.850992° N, 128.590398° E; Figure 10). Measurements and counts in Table 9.

● Paratypes

Vouchers CGRB15866, CGRB15877, CGRB15878 (deposited in the Conservation Genome Resources Bank for Korean Wildlife (CGRB), College of Veterinary Medicine, Seoul National University; field IDs mms3152, mms3904 and mms3909 respectively). CGRB15866 adult collected by Mi-Sook Min, HaeJun Baek and Dong Youn Kim on 6 April 2011 in Geoje-myeon, Geoje-si, Gyeongsang Nam-do, Republic of Korea (34.850992° N, 128.590398° E). CGRB15877 adult collected by Mi-Sook Min on 13 March 2012 in Mundong-dong, Geoje-si, Gyeongsang Nam-do, Republic of Korea (34.857903° N, 128.657295° E). CGRB15878 adult collected by Mi-Sook Min on 13 March 2012 in Mundong-dong, Geoje-si, Gyeongsang Nam-do, Republic of Korea (34.857903° N, 128.657295° E). Measurements and counts in Table 9 illustrations in Figure S3.

● Etymology

The species is endemic to Geoje island in the Republic of Korea, and the name *H. geojeensis* sp. nov. comes from the name of the island where it is restricted. The vernacular name of the species, Geoje salamander, reflects the scientific name of the special, as does its Korean name: 거제도롱뇽 (pronounced: Geoje Dorongnyong).

● Identity, diagnosis and distribution

**Figure 11:**
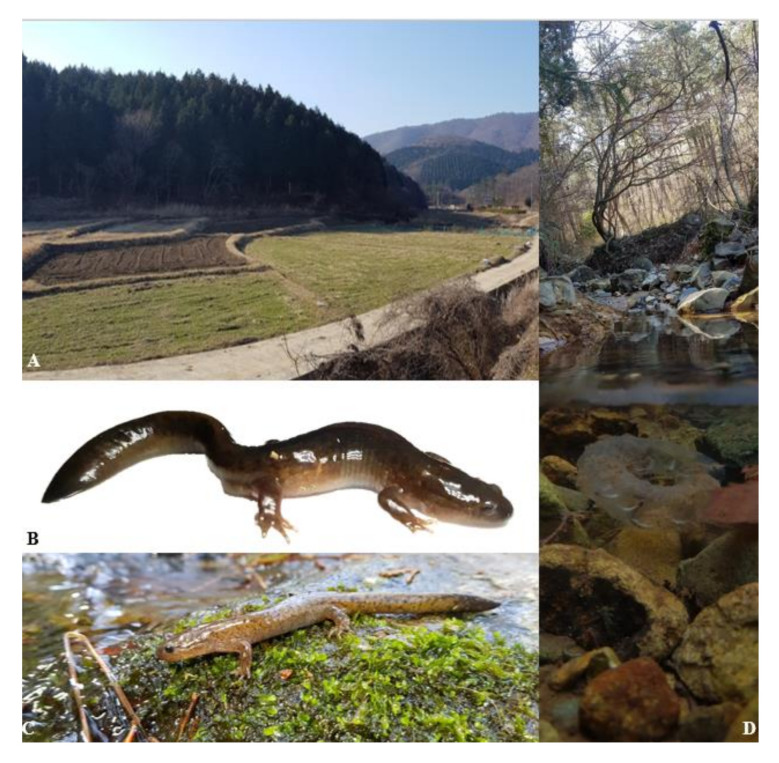
Representative habitat and in life adult, larvae and eggs for *Hynobius geojeensis* sp. nov. (**A**) Habitat during the breeding season (34.842447° N, 128.648362° E on 21 February 2020). (**B**) Adult in situ setting from site (**D**). (**C**) Adult at site (**A**). (**D**) Eggs in situ at breeding site (34.813187° N, 128.617765° E on 18 March 2020).

The species is best identifiable based on range, although it is sympatric with *H. notialis* on the northern half of its range, north of the city of Geoje (Graphical Abstract). *Hynobius geojeensis* is generally broader and longer than *H. notialis* (Table 5 and Table 9), with one exception: limbs are shorted than that of *H. notialis* based on body size (Figure S1) and body ratios (Figure S2). The species can be identified in the area of sympatry if (in mm): SVL < 45.59, TL < 32.67, GA < 22.94, CW < 7.58, HLL < 7.97 and HL < 10.70 (Table 5). The species breeds in natural and agricultural wetlands (Figure 11).

● ZooBank registration

We hereby state that the present paper has been registered to the Official Register of Zoological Nomenclature (ZooBank) under LSID: urn:lsid:zoobank.org:pub:E0E65311-CB76-4AAE-9720-CDF1E6473E71. The new species name *Hynobius geojeensis* sp. nov. has been registered under LSID: urn:lsid:zoobank.org:act:CB0E13D1-4B81-4A59-9548-5A7B6B79856F.

● Nomenclatural acts

The electronic edition of this article conforms to the requirements of the amended International Code of Zoological Nomenclature, and hence the new names contained herein are available under that Code from the electronic edition of this article. This published work and the nomenclatural acts it contains have been registered in ZooBank, the online registration system for the ICZN. The ZooBank LSIDs (Life Science Identifiers) can be resolved and the associated information viewed through any standard web browser by appending the LSID to the prefix “http://zoobank.org/”. The LSID for this publication is: urn:lsid:zoobank.org:pub:E0E65311-CB76-4AAE-9720-CDF1E6473E71. The electronic edition of this work was published in a journal with an ISSN, and has been archived and is available from the following digital repositories: PubMed Central.

#### 3.7.4. *Hynobius perplicatus* sp. nov. Min and Borzée, 2020

Synonymy:

“*Hynobius leechii* (partim): [72]: p. 33; [73]: p. 20”.

“*Hynobius quelpaertensis* (partim): [52]: p. 1166”.

“*Hynobius leechii* Clade HC4: [53]: pp. 25–32; [54]: p. 108; [55]: pp. 165–178”.

● Holotype

**Figure 12:**
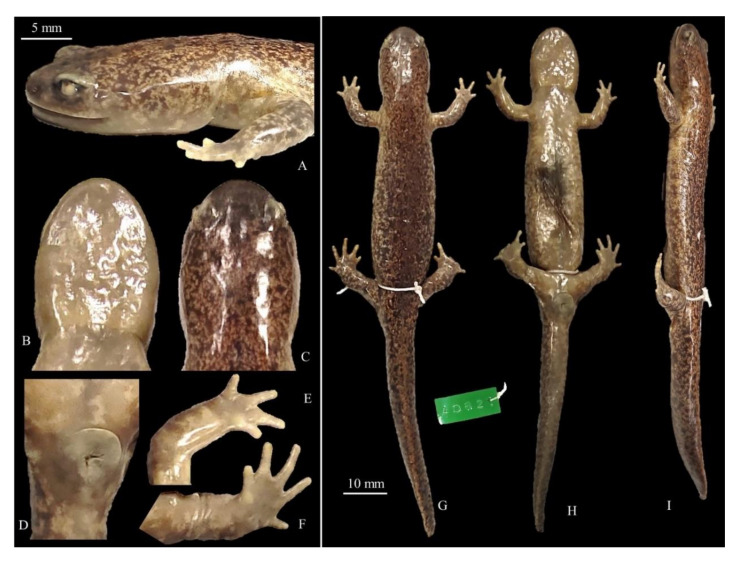
Holotype and details for *Hynobius perplicatus* sp. nov. Voucher CGRB15895 (field ID mms4082); adult collected on 22 March 2012 in Yongdeok-myeon, Uiryeong-gun, South Gyeongsang Province, Republic of Korea (35.348198° N, 128.291133° E). (**A**) Head in lateral view; (**B**) head in ventral view; (**C**) head in dorsal view; (**D**) ventral view of vent; (**E**) planar view of the left hand; (**F**) volar view of the left foot; (**G**) dorsal view; (**H**) ventral view; (**I**) lateral view. (**A**–**F**) match with the 5 mm scale bar and (**G**–**I**). Measurements and counts in Table 9.

Voucher CGRB15895 (deposited in the Conservation Genome Resources Bank for Korean Wildlife (CGRB), College of Veterinary Medicine, Seoul National University; field ID mms4082); adult collected by Mi-Sook Min, HaeJun Baek and DongYoun Kim on 22 March 2012 in Yongdeok-myeon, Uiryeong-gun, Gyeongsang Nam-do, Republic of Korea (35.348198° N, 128.291133° E; Figure 12). Measurements and counts in Table 9.

● Paratypes

Vouchers CGRB15893, CGRB15894, CGRB15896 (deposited in the Conservation Genome Resources Bank for Korean Wildlife (CGRB), College of Veterinary Medicine, Seoul National University; field IDs mms4072, mms4076 and mms4083 respectively). CGRB15893, adult collected by Mi-Sook Min, HaeJun Baek and DongYoun Kim on 22 March 2012 in Yongdeok-myeon, Uiryeong-gun, Gyeongsang Nam-do, Republic of Korea, Republic of Korea (35.348198° N, 128.291133° E). CGRB15894, adult collected by Mi-Sook Min, HaeJun Baek and DongYoun Kim on 22 March 2012in Yongdeok-myeon, Uiryeong-gun, Gyeongsang Nam-do, Republic of Korea, Republic of Korea (35.348198° N, 128.291133° E). CGRB15896, adult collected by Mi-Sook Min, HaeJun Baek and DongYoun Kim on 22 March 2012 in Yongdeok-myeon, Uiryeong-gun, Gyeongsang Nam-do, Republic of Korea, Republic of Korea (35.348198° N, 128.291133° E). Measurements and counts in Table 9 illustrations in Figure S3.

● Etymology

The species occurs on a limited area, sandwiched between the ranges of *H. leechii* and *H. notialis*. The current range is difficult to explain, and is likely related to glaciation patterns, which are so far unresolved, and thus rendering researchers perplexed about the intricate range of this cryptic species. The name *H. perplicatus* sp. nov. comes from this yet unresolved origin of the species’ range, the state of mind of researchers, and the cryptic morphology of the species. “Perplicatus” comes from Latin and means “interlaced, entangled, muddled, intricate, cryptic”. The vernacular name of the species, or Cryptic Uiryeong salamander, reflects the scientific name of the special, as does its Korean name: 숨은의령도롱뇽 (pronounced: Sumeun Uiryeong Dorongnyong).

● Identity, diagnosis and distribution

**Figure 13:**
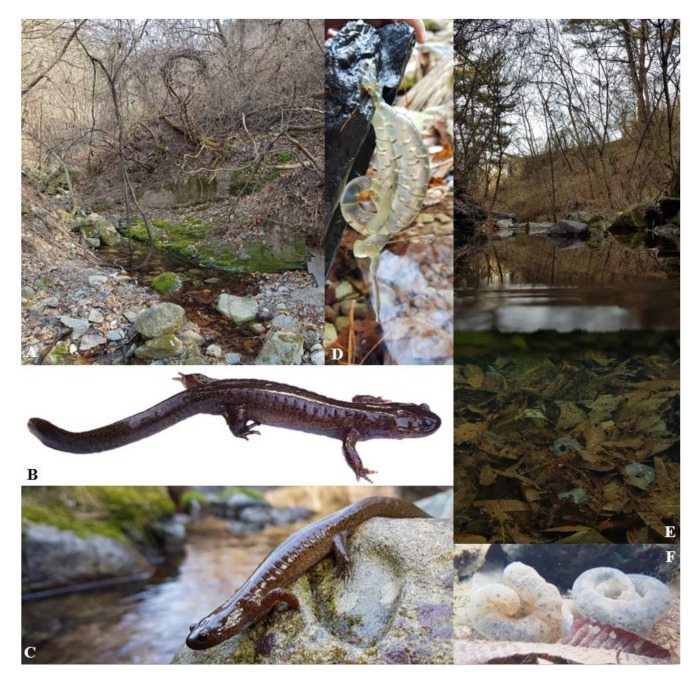
Representative habitat and in life adult, larvae and eggs for *Hynobius perplicatus* sp. nov. (**A**) Habitat during the breeding season (35.368495° N, 128.034015° E on 18 March 2020). (**B**) Adult in situ setting from site (**A**). (**C**) Adult at site (**A**). (**D**) Eggs at site (**A**). (**E**) Eggs in situ at site (**A**). (**F**) Eggs in situ at site (**A**).

To date, the species is known to range from Daebyeong-myeon in Hapcheon on the north west, Sinan-myeon in Sancheong on the south west, Jijeong-myeon in Uiryeong on the south east and Cheongdeok-myeon in Hapcheon on the north east (Graphical Abstract). The species is most easily identified based on range, despite all known populations being sympatric with *H. notialis*. *Hynobius perplicatus* is the largest of all Korean *Hynobius* species (Table 5 and Table 9). While there is no discrete known morphological feature for the species and some individuals may be overlapping with *H. notialis* for some characteristics. *Hynobius perplicatus* is noticeably larger in term or snout-vent-length, tail length, head length, head width and inter-orbital distance. When in sympatry, the species can be identified if (in mm): SVL > 68.21, TL > 62.59, HL > 15.81, HW > 12.78, HL > 4.39 (Table 5). Some characteristics are however of higher value for *H. notialis* and identification needs to include all variables to be reliable (Table 5; Figures S1 and S2). The species breeds in natural and agricultural wetlands (Figure 13).

● ZooBank registration

We hereby state that the present paper has been registered to the Official Register of Zoological Nomenclature (ZooBank) under LSID: urn:lsid:zoobank.org:pub:E0E65311-CB76-4AAE-9720-CDF1E6473E71. The new species name *Hynobius perplicatus* sp. nov. has been registered under LSID: urn:lsid:zoobank.org:act:93D07C4B-1334-4370-8FDB-9BD0A2DD9645.

● Nomenclatural acts

The electronic edition of this article conforms to the requirements of the amended International Code of Zoological Nomenclature, and hence the new names contained herein are available under that Code from the electronic edition of this article. This published work and the nomenclatural acts it contains have been registered in ZooBank, the online registration system for the ICZN. The ZooBank LSIDs (Life Science Identifiers) can be resolved and the associated information viewed through any standard web browser by appending the LSID to the prefix “http://zoobank.org/”. The LSID for this publication is: urn:lsid:zoobank.org:pub:E0E65311-CB76-4AAE-9720-CDF1E6473E71. The electronic edition of this work was published in a journal with an ISSN, and has been archived and is available from the following digital repositories: PubMed Central.

#### 3.7.5. Natural History

Due to the current lack of information on the breeding ecology of the three species described here, we have pooled information on the species’ natural history. Similarly to other Korean *Hynobius* species, the three newly described species reproduce in the still waters of ditches along roads and fields, or in rice paddies during the fallow phase (Figure 9, Figure 11 and Figure 13). Adults and juveniles spend the non-breeding season in the leaf litter of surrounding forests and under shelters such as fallen logs and rocks. Breeding occurs as early as late January, with a peak around late February to early March, generally matching with rain, and late breeders are spawn late March. Males can be found in the water until April. Females may attach the eggs to a substrate when the water is flowing weakly, but also leave eggs unattached at other breeding sites (Figure 9, Figure 11 and Figure 13). Females generally deposit two eggs sacs, one per female’s oviduct, from which larvae emerge once free swimming (Figure 6). Egg sacs are generally curved, even folding back onto an O-shaped loop, although instances of almost straight egg sacs have been found. Larvae develop paired balancers, which resorb when the limbs develop. Larvae are able to walk underwater on the substrate and swim, and metamorphosis occurs before the winter following spawning. The species are not known to be able to overwinter as larvae.

#### 3.7.6. Conservation Status

The assessment presented here follows the IUCN red List criteria and categories (www.iucnredlist.org/resources/redlistguidelines). All *Hynobius* in Korea are facing severe threat, mostly due to habitat destruction and climate change. Most vertebrates have ecological preferences generally matching with those of early humans [74], resulting in early human settlements, and subsequent urbanisation of the habitat such as large alluvial plains favoured by amphibians [75,76], matching with zones of species abundance and pushing species towards secondary habitats. Namely, here the city of Gwangyang, Jinju, Goseong, Tongyeong and Haman (Graphical Abstract) have encroached on the range of the species. In addition, modification of the habitat for rice agriculture has a negative impact on the breeding ecology of the species [77].

All Korean *Hynobius* clades are also impacted by climate change in similar ways, with the exception of sea level rise and habitat salinisation not impacting *H. perplicatus*. Despite calls to limit the current global warming to 1.5 °C above preindustrial levels by the Paris Climate Agreements [78], environmental changes to the ecosystems are estimated to severely impact water resources [79,80]. In addition, the current environmental predictions based on the respect of the Paris Climate Agreements estimate a 2.3 °C increase in air temperature in Asia, and 2.7 °C for North East Asia (peaking at 7.0 °C under a 4 °C scenario if the Paris Climate Agreements are not held; [81]). While critical maximal temperatures are not known for the focal species, experiments for other amphibians show a decrease in fitness through lower speed and resistance to chemical [82]. In addition, the increase in temperature will make rice grows faster [83] and farmers at low latitude in the Republic of Korea take advantage of this phenomenon to alternate barley and rice agriculture in the same fields. This results in an earlier rice plantation, with ploughing while larvae have not metamorphosed yet.

Climate change results in the rise of sea water level, and some sub-populations of the focal species are under the risk of being submerged by sea water [84]. A sea level raise by 60 cm would result in the direct loss of habitat for all species at the exception of *H. perplicatus* (50 to 70 sea water rise under RCP 4.5 scenario by 2100; [85]). In addition, with sea level rise, the coastal habitat will become salinised and will not be adequate for the species [86,87,88]. Moreover, climate change may be expected to result in a northern shift of species range to follow adequate ecological requirements [89], however, and despite the unlikeliness of such dispersal to happen, the current range borders for all three species prevent such shift. Finally the species are under threat because of invasive species such as *Lithobates catesbeianus* [90] emerging diseases such as Batrachochytrids [91,92,93], and the synergy of both type of invasive and pathogens being increasingly released in the environment [94].

*Hynobius yangi*, *H. unisacculus*, *H. geojeensis*, *H. notialis* and *H. perplicatus* have very narrow ranges, <5000 km^2^, fragmented by expenses of saline water and urban areas. The populations have been observed, inferred and projected to be declining in extent of occurrence, area of occupancy, quality of habitat, number of subpopulations and number of mature individuals. As populations sizes are not known, precise dynamics have not been determined and quantitative analyses have not been conducted for any of the species, so the criteria A, C, D and E cannot be used for threat risk estimates.

The range of *H. notialis* is estimated to be <5000 km^2^, which is assigned this threshold to its extent of occurrence (EOO) due to the absence of data on landscape use and presence. The population is known to be present on the mainland and three disconnected islands, and the population is in continuing decline. Therefore, we estimate *H. notialis* to be in the Endangered category following the criteria of the IUCN Red List of Threatened Species under the criteria B1ab(i,ii,iii,iv,v).

The range of *H. geojeensis* is estimated to be <500 km^2^, which is assigned this threshold by the EOO for the species. In addition, the area of occupancy (AOO) of the species, based on island area as a proxy for range is <100 km^2^. The population is known to be present on the island only, and it is in continuing decline. Therefore, we estimate *H. notialis* to be in the Critically Endangered category following the criteria of the IUCN Red List of Threatened Species under the criteria B1ab(i,ii,iii,iv,v).

The range of *H. perplicatus* is estimated to be <1000 km^2^, which is assigned this threshold to the EOO due to the absence of data on landscape use and presence. The population is known to be present at two connected sites, but it is also likely to occur in linked valleys. The population is in continuing decline. Therefore, we estimate *H. perplicatus* to be in the Endangered category following the criteria of the IUCN Red List of Threatened Species under the criteria B1ab(i,ii,iii,iv,v).

The range of *H. yangi* is estimated to be <1000 km^2^, although the ancestral range may have reached 1200 km^2^, now reduced by about 150 km^2^ by the city of Busan and about 100 km^2^ by the city of Ulsan. Therefore, the species EOO is <1000 km^2^ and its AOO circa half of this value based on landscape use and the heavy urbanisation of the landscape within the range of the species. The population is in continuous decline, with several extirpated population due to development and the remaining sub-populations are divided into a maximum of four disconnected areas due to urban tracts and motorways. While threats are well understood, they have not ceased, and the range of the species is constantly decreasing due to expansion and urbanisation from the cities of Busan and Ulsan. Therefore, we estimate *H. yangi* to be in the Endangered category following the criteria of the IUCN Red List of Threatened Species under the criteria B1ab(i,ii,iii,iv,v).

The range of *H. unisacculus* is estimated to be <2500 km^2^, which is assigned this threshold by the EOO for the species. In addition, the AOO of the species is <2000 km^2^ based on landscape and habitat that can be used by the species. The population is severely fragmented due to the species distribution on islands, peninsula and the mainland, and urban areas and highways. In addition, a continuous decline is observed for EOO, AOO, and extant and quality of the habitat. Therefore, we estimate *H. unisacculus* to be in the Vulnerable category following the criteria of the IUCN Red List of Threatened Species under the criteria B1ab(i,ii,iii).

The range of *H. quelpaertensis* is estimated to be <20,000 km^2^, which is assigned this value to the species EOO, and an unknown AOO due to the absence of data on the fine scale distribution and habitat use by the species. In addition, the population is fragmentated due to the coastal and continental distribution of the species. The habitat is continuously decreasing in quality and extent, resulting in decreasing in EOO and AOO. The threats to *H. quelpaertensis* are known but not mitigated. Therefore, we estimate *H. quelpaertensis* to be in the Vulnerable category following the criteria of the IUCN Red List of Threatened Species under the criteria B1ab(i,ii,iii).

Finally, the range of *H. leechii* is estimated to be significantly wider than 20,000 km^2^, and the northern boundary of the species range is not yet defined. Consequently, the EOO of the species is larger than the threshold used to trigger any of the threatened categories following the criteria of the IUCN Red List of Threatened Species. Therefore, we estimate *H. leechii* to be falling in the category Least Concern following the criteria of the IUCN Red List of Threatened Species.

## 4. Discussion

Here, we demonstrated that the morphology of *Hynobius* sp. in the Republic of Korea is greatly variable based on both biotic and abiotic variables. All three of our hypotheses were supported. Namely, each of the seven phylogenetic *Hynobius* clade had a significantly different morphology, despite the significant island and patry effects. The islandic populations were significantly smaller than those on the mainland when corrected for inter clade variation. Finally, once corrected for clade and island effect, patry was also found to significantly impacting the morphology of the genus. We conclude by describing the three candidate species and caution against relying on morphology only for species description, despite being the only tool available for the past 100+ years, as the true diversity of species can be overlooked because of the significant island and patry effects on morphology.

While the genetic differences between the seven clades have been known and demonstrated prior to this study (Figure 7; [42,51,52]), we could not predict the presence of corresponding morphological differences. The distribution of all species except for *H. leechii* is geographically restricted to the same ecoregion, with consistent but not widespread contact zones. As a result, the ecological requirements of the species are not expected to be largely different, although it would be a scientifically valid endeavour to test such hypothesis, and thus the morphology of the species may not have differentiated to adapt to specific ecological requirements, or non-overlapping niches. However, our results show a difference in morphology between clades, maybe due to drift from the morphology of the common ancestor after isolation. While there is a significant difference between species, these are not easily visible to the untrained observer, and can be explained by a community-wide character displacement [95,96], linked to similar requirement and the absence of competition due to the non-overlapping niches [9]. This pattern was first described as a coevolutionary morphological response within “ecological guild” [97], where species see their morphology tend towards equal size ratio, likely to avoid interspecific competition [10,96]. Therefore, in the absence of competition because of sympatry, there is no selection for morphological shift, and the best adapted morphology is likely to be very similar between clades. Here the significant variations in morphology may be local adaptations to specific landscape features. For instance, *H. perplicatus* occurs in landscapes with larger water-bodies that in turn may enable a comparatively larger body size. In contrast *H. geojeensis* is smaller than the others species, possibly because the island is relatively dry, with few springs available, and a smaller body size is likely to help breeding is shallow water bodies. In addition, the difference in species communities on which larvae can prey may affect the morphology of the species. For instance, the absence of *Rana huanrenensis* on the range of *H. geojeensis* only [98] may result in a partial shift in diet, and consequently in morphology. We recommend further ecological studies on this topic to confirm this hypothesis.

In contrast, the more important morphological difference in areas of sympatry was such as hypothesised, a likely result of ecological character displacement to decrease competition [19,20,21,22,23,99]. An interesting point here is the shift in morphology for the species that have the largest number of sympatric areas. For instance, *H. unisacculus* is generally smaller than all other species, likely because it is one of the two species sharing the largest number of sympatric areas. The other species is *H. notialis*, which displays different variations of morphology in function of the species it is sharing a sympatric area with (Figure 5). For instance, when in allopatry the average TL of *H. notialis* (main loading PC significantly different between clades) is close to that of all seven species. However, once in sympatry with *H. unisacculus*, the TL of *H. notialis* drastically drops, to the point that the median value is below the third quartile, highlighting a clear segregation between the two species, and a potential ecological character displacement as TL is then different between the two species. When in sympatry with *H. geojeensis*, the TL median value of *H. notialis* is higher than for allopatric populations, although the third quartile is still lower than the lowest value for *H. geojeensis*. When in sympatry with *H. perplicatus*, the median is different as well, although higher than that of the sympatric species. This pattern demonstrates that while the morphology of the species is generally consistent between sub-populations, local discrete pressure can result in strong divergences.

While the impact of the island effect is not questionable, it has not been as largely documented for amphibians as for mammals or other charismatic species. It is therefore interesting to demonstrate its impacts on *Hynobius* sp. on the Korean Peninsula. The size reduction experienced by the species on the islands is in line with that of other amphibians, and matching with the size reduction likely happening to *Bufo gargarizans* on other Korean islands [100]. However, the size reduction is opposite to that expressed by islandic populations of *Dryophytes japonicus* on Jeju Island [28,101], one of the southernmost and largest island where the focal species are distributed. It is so far not known why the islandic populations are smaller, although it could be related to the decreased abundance of water bodies, and being a non-directional shift in morphology.

Finally, the description of three new *Hynobius* species solves the taxonomic question regarding these three clades. It, however, raises a new set of questions related to their conservation. One of the three new species is distributed in such a narrow range that we recommend it becomes listed as Critically Endangered by the IUCN Red List of species, and we recommend listing the two other species as Endangered—and Endangered Class II by the government of the Republic of Korea. A strong emphasis should be brought to the conservation status of *H. geojeensis*, as the species is not known from more five localities, some of which are being heavily modified by human activities. We therefore recommend protection of the habitat instead of ex-situ breeding and release, as boosting the population size will not provide additional breeding habitat.

## 5. Conclusions

The results presented here highlight the relationships between morphological adaptations and habitat, and variations based on multiples factors. We have demonstrated that individuals are larger on the mainland than on islands, and that within a species, populations in sympatry are different from those in allopatry. Besides the multiple intra-clade variations, we also demonstrated significant morphological differences between the seven clades studies here, and we described *Hynobius geojeensis*, *Hynobius notialis* and *Hynobius perplicatus*. All new species are restricted to very narrow ranges and conservation efforts are paramount for the survival of the species.

## Figures and Tables

**Table 1:** Correlation table for all morphological variables for *Hynobius* sp. specimens. We conducted a Person correlation test for all morphological variables included in the analyses (*n* = 293). Each variable was measured three times and averaged, and data are presented in the form of a ratio with the focal variables divided by SVL. Significant variables are in bold, and abbreviations are explained in text and in Table 3.

Variables		GA	CW	FLL	HLL	HL	HW	EL	IN	ON	IO	OR	IC
TL	R	0.07	0.51	0.24	0.36	0.11	0.37	0.06	0.29	0.06	0.30	0.00	0.14
	*p*	0.269	**<0.001**	**<0.001**	**<0.001**	0.052	**<0.001**	0.285	**<0.001**	0.277	**<0.001**	0.951	**0.020**
GA	R		−0.05	0.03	−0.06	−0.24	−0.10	−0.13	−0.05	−0.12	−0.08	−0.05	−0.04
	*p*		0.388	0.654	0.284	**<0.001**	0.076	**0.023**	0.335	**0.033**	0.158	0.400	0.457
CW	R			0.44	0.45	0.22	0.60	0.19	0.29	0.09	0.36	0.13	0.25
	*p*			**<0.001**	**<0.001**	**<0.001**	**<0.001**	**<0.001**	**<0.001**	0.124	**<0.001**	**0.020**	**<0.001**
FLL	R				0.49	0.05	0.39	0.22	0.19	0.09	0.20	0.13	0.15
	*p*				**<0.001**	0.421	**<0.001**	**<0.001**	**<0.001**	0.093	**<0.001**	**0.020**	**0.007**
HLL	R					0.15	0.34	0.20	0.25	0.13	0.21	0.13	0.16
	*p*					**0.005**	**<0.001**	**<0.001**	**<0.001**	**0.018**	**<0.001**	**0.021**	**0.004**
HL	R						0.38	0.38	0.25	0.27	0.30	0.33	0.38
	*p*						**<0.001**	**<0.001**	**<0.001**	**<0.001**	**<0.001**	**<0.001**	**<0.001**
HW	R							0.40	0.39	0.26	0.43	0.18	0.39
	*p*							**<0.001**	**<0.001**	**<0.001**	**<0.001**	**0.001**	**<0.001**
EL	R								0.28	0.25	0.27	0.31	0.33
	*p*								**<0.001**	**<0.001**	**<0.001**	**<0.001**	**<0.001**
IN	R									0.19	0.39	0.23	0.24
	*p*									**0.001**	**<0.001**	**<0.001**	**<0.001**
ON	R										0.21	0.35	0.30
	*p*										**<0.001**	**<0.001**	**<0.001**
IO	R											0.26	0.36
	*p*											**<0.001**	**<0.001**
OR	R												0.31
	*p*												**<0.001**

**Table 2:** Comparison of principal component analyses. We compared the percentages of variance expressed by the principal components based on two datasets. The first dataset includes the variable TL, resulting in all individuals without the measurement for TL being excluded from the dataset (293 individuals; 13 variables). The second dataset excludes the variables TL, so all individuals were included in the analysis (329 individuals; 12 variables). The PCs were selected if the eigenvalues were >1, based on varimax rotations. The total loading cumulative percentage is in bold, and we retained the dataset including TL.

PCs	Eigenvalue	Percentage of Variance	Cumulative Percentage
Including Tail Length			
PC1	3.98	30.67	30.67
PC2	1.77	13.67	44.34
PC3	1.03	7.90	52.24
PC4	1.02	7.84	**60.09**
Excluding Tail Length			
PC1	3.83	31.90	31.90
PC2	1.56	13.02	44.92
PC3	1.03	8.56	**53.49**

**Table 3:** Component matrix with the loading factors of each variable into the PCs. The factors were extracted with principal component analysis under a varimax rotation with Kaiser normalisation. Rotation converged in 7 iterations. The representative PCs are in bold. The PCs represent the following traits. PC1: body structure; PC2: head structure; PC3: limbs; PC4: GA. The value used as the cut-off was 0.47, and loading variables are in bold. * HL loaded similarly into two PCs, secondarily into PC4.

Variable		PC1	PC2	PC3	PC4
TL	Tail length	**0.689**	−0.140	0.292	0.144
GA	Gleno-acetabular distance	0.045	−0.024	−0.027	**0.922**
CW	Chest width	**0.668**	−0.025	0.491	−0.098
FLL	Forelimb length	0.118	0.086	**0.834**	0.059
HLL	Hindlimb length	0.224	0.135	**0.774**	−0.064
HL	Head length	0.367	**0.472**	−0.067	−0.460 *
HW	Head width	**0.679**	0.228	0.297	−0.197
EL	Eye length	0.180	**0.594**	0.189	−0.204
IN	Inter-nostril distance	**0.569**	0.248	0.053	0.027
ON	Orbitonarial distance	0.040	**0.732**	0.057	0.005
IO	Interorbital distance	**0.685**	0.248	0.016	−0.060
OR	Orbitorostral distance	0.046	**0.774**	0.120	0.019
IC	Intercanthal distance	0.424	**0.562**	−0.123	−0.041

**Table 4:** Tukey’s post-hoc test. We used these tests to assess for pair-wise morphometric differences between clades. All species sharing boundaries had at least one significant significantly different PC. (I-J) is the mean difference. Sp. nov. is abbreviated as sp. n. and all significant variables are in bold.

Clade (I)	Clade (J)	PC1			PC2			PC3			PC4		
		(I-J)	SE	*p*	(I-J)	SE	*p*	(I-J)	SE	*p*	(I-J)	SE	*p*
*H. leechii*	*H. quelpaertensis*	0.21	0.20	0.941	0.49	0.22	0.272	0.25	0.22	0.916	0.17	0.23	0.989
	*H. yangi*	0.34	0.24	0.799	0.13	0.27	0.999	0.81	0.27	**0.045**	0.48	0.28	0.625
	*H. unisacculus*	1.12	0.18	**<0.001**	0.03	0.20	1.000	0.84	0.20	**0.001**	0.02	0.21	1.000
	*H. notialis* sp. n	0.50	0.16	**0.037**	0.58	0.18	**0.022**	0.37	0.18	0.382	−0.11	0.19	0.997
	*H. geojeensis* sp. n	−0.15	0.23	0.994	−0.09	0.25	1.000	0.61	0.25	0.199	0.02	0.26	1.000
	*H. perplicatus* sp. n	−0.58	0.19	**0.037**	0.86	0.21	**0.001**	−0.08	0.21	1.000	0.03	0.22	1.000
*H. quelpaert-ensis*	*H. leechii*	−0.21	0.20	0.941	−0.49	0.22	0.272	−0.25	0.22	0.916	−0.17	0.23	0.989
	*H. yangi*	0.14	0.25	0.998	−0.36	0.28	0.845	0.56	0.28	0.392	0.30	0.29	0.941
	*H. unisacculus*	0.92	0.19	**<0.001**	−0.46	0.21	0.320	0.59	0.21	0.077	−0.16	0.22	0.992
	*H. notialis* sp. n	0.29	0.17	0.609	0.10	0.19	0.999	0.12	0.19	0.995	−0.28	0.20	0.785
	*H. geojeensis* sp. n	−0.36	0.23	0.726	−0.58	0.26	0.278	0.36	0.26	0.809	−0.15	0.27	0.998
	*H. perplicatus* sp. n	−0.79	0.20	**0.002**	0.37	0.22	0.617	−0.33	0.22	0.743	−0.14	0.23	0.996
*H. yangi*	*H. leechii*	−0.34	0.24	0.799	−0.13	0.27	0.999	−0.81	0.27	**0.045**	−0.48	0.28	0.625
	*H. quelpaertensis*	−0.14	0.25	0.998	0.36	0.28	0.845	−0.56	0.28	0.392	−0.30	0.29	0.941
	*H. unisacculus*	0.78	0.24	**0.021**	−0.09	0.26	1.000	0.03	0.26	1.000	−0.46	0.28	0.641
	*H. notialis* sp. n	0.16	0.22	0.992	0.46	0.25	0.516	−0.44	0.25	0.568	−0.59	0.26	0.264
	*H. geojeensis* sp. n	−0.49	0.28	0.552	−0.22	0.30	0.992	−0.20	0.30	0.994	−0.46	0.32	0.778
	*H. perplicatus* sp. n	−0.92	0.24	**0.003**	0.73	0.27	0.098	−0.89	0.27	**0.018**	−0.45	0.28	0.695
*H. unisac-culus*	*H. leechii*	−1.12	0.18	**<0.001**	−0.03	0.20	1.000	−0.84	0.20	**0.001**	−0.02	0.21	1.000
	*H. quelpaertensis*	−0.92	0.19	**<0.001**	0.46	0.21	0.320	−0.59	0.21	0.077	0.16	0.22	0.992
	*H. yangi*	−0.78	0.24	**0.021**	0.09	0.26	1.000	−0.03	0.26	1.000	0.46	0.28	0.641
	*H. notialis* sp. n	−0.62	0.16	**0.002**	0.55	0.17	**0.026**	−0.47	0.17	0.098	−0.13	0.18	0.992
	*H. geojeensis* sp. n	−1.28	0.22	**<0.001**	−0.12	0.25	0.999	−0.23	0.25	0.964	0.00	0.26	1.000
	*H. perplicatus* sp. n	−1.71	0.18	**<0.001**	0.82	0.20	**0.001**	−0.92	0.20	**<0.001**	0.01	0.21	1.000
*H. notialis* sp. nov.	*H. leechii*	−0.50	0.16	**0.037**	−0.58	0.18	**0.022**	−0.37	0.18	0.382	0.11	0.19	0.997
	*H. quelpaertensis*	−0.29	0.17	0.609	−0.10	0.19	0.999	−0.12	0.19	0.995	0.28	0.20	0.785
	*H. yangi*	−0.16	0.22	0.992	−0.46	0.25	0.516	0.44	0.25	0.568	0.59	0.26	0.264
	*H. unisacculus*	0.62	0.16	**0.002**	−0.55	0.17	0.026	0.47	0.17	0.098	0.13	0.18	0.992
	*H. geojeensis* sp. n	−0.65	0.21	**0.029**	−0.67	0.23	**0.050**	0.24	0.23	0.947	0.13	0.24	0.998
	*H. perplicatus* sp. n	−1.08	0.16	**<0.001**	0.27	0.18	0.738	−0.45	0.18	0.165	0.14	0.19	0.989
*H. geojeensis* sp. nov.	*H. leechii*	0.15	0.23	0.994	0.09	0.25	1.000	−0.61	0.25	0.199	−0.02	0.26	1.000
	*H. quelpaertensis*	0.36	0.23	0.726	0.58	0.26	0.278	−0.36	0.26	0.809	0.15	0.27	0.998
	*H. yangi*	0.49	0.28	0.552	0.22	0.30	0.992	0.20	0.30	0.994	0.46	0.32	0.778
	*H. unisacculus*	1.28	0.22	**<0.001**	0.12	0.25	0.999	0.23	0.25	0.964	0.00	0.26	1.000
	*H. notialis* sp. n	0.65	0.21	**0.029**	0.67	0.23	**0.050**	−0.24	0.23	0.947	−0.13	0.24	0.998
	*H. perplicatus* sp. n	−0.43	0.23	0.493	0.95	0.25	**0.004**	−0.69	0.25	0.097	0.01	0.26	1.000
*H. perplicatus* sp. nov.	*H. leechii*	0.58	0.19	**0.037**	−0.86	0.21	**0.001**	0.08	0.21	1.000	−0.03	0.22	1.000
	*H. quelpaertensis*	0.79	0.20	**0.002**	−0.37	0.22	0.617	0.33	0.22	0.743	0.14	0.23	0.996
	*H. yangi*	0.92	0.24	**0.003**	−0.73	0.27	0.098	0.89	0.27	**0.018**	0.45	0.28	0.695
	*H. unisacculus*	1.71	0.18	**<0.001**	−0.82	0.20	**0.001**	0.92	0.20	**<0.001**	−0.01	0.21	1.000
	*H. notialis* sp. n	1.08	0.16	**<0.001**	−0.27	0.18	0.738	0.45	0.18	0.165	−0.14	0.19	0.989
	*H. geojeensis* sp. n	0.43	0.23	0.493	−0.95	0.25	**0.004**	0.69	0.25	0.097	−0.01	0.26	1.000

**Table 5:** Details of morphometric variables. Mean, standard deviation, minimum and maximum values for each morphological variable for each species. These values are averaged per species.

Items		SVL	TL	GA	CW	FLL	HLL	HL	HW	EL	IN	ON	IO	OR	IC
*Hynobius leechii*	Mean	57.14	48.14	29.13	10.57	13.62	15.76	13.54	10.60	3.25	3.78	2.49	3.66	4.08	5.29
	SD	7.01	8.23	3.88	1.48	1.90	2.27	1.65	1.11	0.33	0.57	0.43	0.61	0.49	0.59
	Min	44.81	31.85	20.94	6.98	10.33	9.29	10.65	8.45	2.70	2.61	1.71	2.54	2.93	4.33
	Max	70.01	69.81	37.41	14.16	17.24	21.68	17.82	13.35	3.96	5.05	3.99	5.19	5.12	6.58
*Hynobius quelpaertensis*	Mean	53.76	43.86	27.69	9.74	12.13	14.58	12.73	10.15	2.93	3.28	2.24	3.20	3.60	4.83
	SD	7.52	7.99	4.38	1.13	1.59	1.96	1.51	1.20	0.34	0.54	0.31	0.42	0.56	0.62
	Min	38.51	30.35	17.57	7.32	9.15	10.25	9.30	7.62	2.22	2.40	1.51	2.25	2.72	3.84
	Max	70.24	65.57	36.86	12.12	15.03	18.80	16.00	13.20	3.47	4.45	2.78	4.25	4.74	6.28
*Hynobius yangi*	Mean	52.46	39.30	26.58	9.27	11.31	13.62	12.56	9.70	3.01	3.29	2.06	3.18	3.62	4.89
	SD	7.31	6.92	4.55	1.14	1.76	2.07	1.48	1.23	0.33	0.55	0.27	0.60	0.40	0.71
	Min	33.55	21.34	16.30	5.85	7.57	8.52	8.97	6.82	2.33	1.99	1.64	1.93	2.71	3.31
	Max	61.73	48.62	33.18	11.03	13.79	16.85	14.49	12.23	3.46	4.69	2.67	4.37	4.64	6.39
*Hynobius unisacculus*	Mean	49.10	36.33	25.02	8.13	10.36	12.44	11.20	8.49	2.78	2.99	1.99	2.78	3.44	4.39
	SD	5.36	6.11	3.42	1.03	1.51	1.83	1.20	0.90	0.28	0.48	0.20	0.43	0.42	0.56
	Min	32.91	23.91	16.04	5.80	6.95	8.37	7.84	6.38	2.29	2.22	1.58	1.89	2.66	2.65
	Max	61.29	50.15	31.92	10.72	13.76	17.57	14.00	10.83	3.45	4.82	2.52	3.89	4.68	6.19
*Hynobius notialis* sp. nov.	Mean	54.78	44.17	28.80	9.94	12.08	14.68	12.69	9.71	3.06	3.22	2.13	3.21	3.65	4.96
	SD	5.53	5.87	5.22	1.22	1.69	1.93	1.08	1.05	0.28	0.36	0.33	0.43	0.54	0.62
	Min	45.59	32.67	22.94	7.58	7.97	9.34	10.70	5.86	2.39	2.44	1.29	2.23	1.73	4.04
	Max	68.21	62.59	62.63	14.66	15.98	21.06	15.81	12.78	3.86	4.31	3.54	4.39	5.41	7.77
*Hynobius geojeensis* sp. nov.	Mean	52.02	45.06	26.50	9.54	11.29	13.94	12.41	9.60	2.92	3.36	2.34	3.24	3.78	4.92
	SD	7.37	6.41	4.54	1.35	1.68	2.32	1.44	1.26	0.41	0.41	0.44	0.44	0.48	0.73
	Min	32.95	26.58	17.29	5.88	7.15	7.71	8.37	6.61	2.38	2.25	1.68	2.44	2.45	3.18
	Max	67.31	55.61	34.84	12.06	14.38	19.36	16.00	12.56	4.04	4.20	3.49	4.04	5.03	6.44
*Hynobius perplicatus* sp. nov.	Mean	61.46	53.29	31.67	12.24	14.45	17.49	14.42	11.75	3.38	4.06	2.37	4.00	4.16	5.65
	SD	3.95	5.57	2.50	0.99	1.63	1.77	0.86	0.89	0.29	0.57	0.31	0.51	0.41	0.67
	Min	54.32	42.58	27.27	10.24	9.94	11.28	12.46	10.15	2.63	1.28	1.87	3.04	3.28	3.61
	Max	70.25	68.39	37.41	13.92	18.09	20.69	16.30	14.64	3.88	4.90	3.08	5.41	4.87	6.99

**Table 6:** Results of the general linear mixed model testing for the impact of the island effect. We used the islandic variable as the dependent variable—scaled with the sympatric variable to remove bias; and we offset the covariates with the variable clade to correct for the impact of inter-clade variations. The covariates in this analysis were the four PCs extracted from the PCA, and the analysis was run under a main effect model. Significant variables are in bold.

Items	Tests			Parameters Estimates	
	Wald χ^2^	df	*p*	B	SE
PC1	4.74	1	**0.029**	−0.32	0.15
PC2	10.49	1	**0.001**	0.40	0.12
PC3	11.46	1	**0.001**	−0.38	0.11
PC4	0.13	1	0.715	0.05	0.14

**Table 7:** Results of the general linear mixed model testing for the impact of the patry. We used the patry variable as the dependent variable, scaled with the variable “sympatric with” to remove bias, and we offset the covariates with the variable clade to correct for the impacts of inter-clade variations. The covariates in this analysis were the four PCs extracted from the PCA, and the analysis was run under a main effect model. Significant variables are in bold.

Items	Tests			Parameters Estimates	
	Wald χ^2^	df	*p*	B	SE
PC1	4.75	1	**0.029**	−0.28	0.13
PC2	0.16	1	0.687	0.05	0.12
PC3	8.22	1	**0.004**	−0.31	0.11
PC4	3.92	1	**0.048**	−0.26	0.13

**Table 8:** Tukey’s post-hoc test. We used this test to determine the significant differences within clades in relation with species in sympatry. Significant variables are in bold.

			Mean Difference (I-J)	SE	*p*
PC1	*Hynobius unisacculus*	*H. notialis* sp. nov.	−0.71	0.25	**0.034**
		*H. geojeensis* sp. nov	−0.87	0.39	0.129
		*H. perplicatus* sp. nov.	−0.55	0.42	0.565
	*Hynobius notialis* sp. nov.	*H. unisacculus*	0.71	0.25	**0.034**
		*H. geojeensis* sp. nov	−0.16	0.34	0.963
		*H. perplicatus* sp. nov.	0.16	0.37	0.975
	*Hynobius geojeensis* sp. nov	*H. unisacculus*	0.87	0.39	0.129
		*H. notialis* sp. nov.	0.16	0.34	0.963
		*H. perplicatus* sp. nov.	0.32	0.48	0.909
	*Hynobius perplicatus* sp. nov.	*H. notialis* sp. nov.	0.55	0.42	0.565
		*H. notialis* sp. nov.	−0.16	0.37	0.975
		*H. geojeensis* sp. nov	−0.32	0.48	0.909
PC2	*Hynobius unisacculus*	*H. notialis* sp. nov.	−0.11	0.32	0.986
		*H. geojeensis* sp. nov	0.04	0.49	1.000
		*H. perplicatus* sp. nov.	0.68	0.53	0.576
	*Hynobius notialis* sp. nov.	*H. unisacculus*	0.11	0.32	0.986
		*H. geojeensis* sp. nov	0.15	0.42	0.984
		*H. perplicatus* sp. nov.	0.79	0.47	0.341
	*Hynobius geojeensis* sp. nov	*H. unisacculus*	−0.04	0.49	1.000
		*H. notialis* sp. nov.	−0.15	0.42	0.984
		*H. perplicatus* sp. nov.	0.64	0.60	0.713
	*Hynobius perplicatus* sp. nov.	*H. notialis* sp. nov.	−0.68	0.53	0.576
		*H. notialis* sp. nov.	−0.79	0.47	0.341
		*H. geojeensis* sp. nov	−0.64	0.60	0.713
PC3	*Hynobius unisacculus*	*H. notialis* sp. nov.	−0.70	0.34	0.180
		*H. geojeensis* sp. nov	−0.48	0.53	0.796
		*H. perplicatus* sp. nov.	−1.29	0.57	0.119
	*Hynobius notialis* sp. nov.	*H. unisacculus*	0.70	0.34	0.180
		*H. geojeensis* sp. nov	0.22	0.46	0.965
		*H. perplicatus* sp. nov.	−0.59	0.50	0.647
	*Hynobius geojeensis* sp. nov	*H. unisacculus*	0.48	0.53	0.796
		*H. notialis* sp. nov.	−0.22	0.46	0.965
		*H. perplicatus* sp. nov.	−0.81	0.65	0.599
	*Hynobius perplicatus* sp. nov.	*H. notialis* sp. nov.	1.29	0.57	0.119
		*H. notialis* sp. nov.	0.59	0.50	0.647
		*H. geojeensis* sp. nov	0.81	0.65	0.599
PC4	*Hynobius unisacculus*	*H. notialis* sp. nov.	−0.38	0.28	0.538
		*H. geojeensis* sp. nov	−0.87	0.43	0.197
		*H. perplicatus* sp. nov.	−0.34	0.47	0.884
	*Hynobius notialis* sp. nov.	*H. unisacculus*	0.38	0.28	0.538
		*H. geojeensis* sp. nov	−0.50	0.38	0.555
		*H. perplicatus* sp. nov.	0.03	0.42	1.000
	*Hynobius geojeensis* sp. nov	*H. unisacculus*	0.87	0.43	0.197
		*H. notialis* sp. nov.	0.50	0.38	0.555
		*H. perplicatus* sp. nov.	0.53	0.53	0.754
	*Hynobius perplicatus* sp. nov.	*H. notialis* sp. nov.	0.34	0.47	0.884
		*H. notialis* sp. nov.	−0.03	0.42	1.000
		*H. geojeensis* sp. nov	−0.53	0.53	0.754

**Table 9:** Morphometric data for holotypes and paratypes. Each variable was measured three times and averaged. One cell is annotated as Non Available because the individual had its finger cut off for DNA extraction in previous work, and based on the threats to the species, a missing finger did not justify killing an additional individual.

Species	*Hynobius notialis* sp. nov.				*Hynobius geojeensis* sp. nov.				*Hynobius perplicatus* sp. nov.			
Voucher ID	CGRB15873	CGRB15859	CGRB15870	CGRB15884	CGRB15863	CGRB15866	CGRB15877	CGRB15878	CGRB15895	CGRB15893	CGRB15894	CGRB15896
Field ID	mms3897	mms3136	mms3894	mms4036	mms3149	mms3152	mms3904	mms3909	mms4082	mms4072	mms4076	mms4083
Type	Holotype	Paratype	Paratype	Paratype	Holotype	Paratype	Paratype	Paratype	Holotype	Paratype	Paratype	Paratype
Series	Left	Left	Right	Left	Right	Right	Right	Front Right Back Left	Left	Left	Right	Left
SVL	56.44	49.98	50.29	60.06	52.88	58.18	55.05	67.31	69.66	62.29	63.94	70.25
TL	43.07	42.75	39.25	47.99	48.20	55.61	48.92	48.81	55.28	56.69	49.67	62.37
GA	29.20	24.86	25.74	32.32	27.69	32.47	27.37	34.84	37.41	31.87	32.94	36.69
CW	8.65	10.21	8.66	10.83	10.04	10.23	10.64	12.06	12.13	11.93	11.38	13.92
FLL	10.15	10.87	12.43	15.98	12.40	13.55	11.79	14.38	16.28	13.40	12.77	18.09
HLL	13.91	13.24	14.33	20.13	15.82	16.42	15.56	19.36	18.57	18.52	15.38	20.69
HL	12.79	11.86	12.34	12.97	12.59	13.16	12.70	16.00	15.71	15.07	14.61	16.18
HW	9.16	9.55	9.21	5.86	9.71	10.80	10.04	12.56	11.21	11.95	10.48	13.11
EL	2.89	3.08	3.11	3.18	2.45	3.10	3.33	4.04	3.37	3.50	3.13	3.86
IN	3.36	2.97	3.10	3.18	3.46	3.63	4.20	4.20	4.04	4.30	3.78	4.63
ON	1.95	2.43	1.81	2.11	2.32	3.49	2.57	3.02	1.99	2.01	2.33	2.47
IO	2.68	2.98	2.75	3.05	3.12	3.55	3.67	4.04	3.42	4.03	5.27	4.15
OR	3.75	3.53	3.84	4.13	4.05	4.16	4.22	5.03	4.36	4.59	4.37	4.55
IC	4.64	4.62	4.67	5.12	5.11	5.49	5.14	6.01	5.70	5.67	5.51	6.09
1-FL	1.51	1.99	1.92	2.01	2.49	1.61	1.89	1.31	1.60	2.09	2.01	2.27
2-FL	1.99	2.83	2.70	3.40	2.69	2.08	2.49	3.37	3.07	2.25	2.69	2.84
3- FL	2.94	2.61	2.61	3.60	2.70	1.39	3.37	3.19	2.84	2.92	2.59	4.00
4-FL	1.85	1.63	1.95	2.13	1.49	1.47	1.82	1.89	2.00	1.55	1.16	1.98
1-TL	1.45	1.54	2.34	2.22	2.17	1.55	1.46	2.44	1.92	2.41	2.24	2.29
2-TL	2.76	3.36	3.46	3.86	2.87	2.87	2.95	3.93	3.39	3.50	4.10	3.75
3-TL	3.66	4.12	4.02	4.12	4.42	4.00	NA	4.53	4.41	4.78	3.93	4.53
4-TL	2.73	2.84	3.38	2.79	3.37	2.47	2.52	3.09	2.85	4.61	2.34	4.15
5-TL	1.82	1.79	1.70	2.03	2.05	2.02	1.92	1.81	2.00	2.26	1.36	3.19
MTH	5.65	6.28	5.62	7.39	6.66	6.94	7.88	8.17	7.08	8.20	5.97	7.85
MTW	4.04	4.29	3.89	5.46	2.79	5.03	3.81	5.79	5.51	5.15	4.01	5.08
MAXTH	5.12	5.99	5.50	7.64	5.44	7.06	6.84	8.26	6.85	8.59	5.93	8.97
CGN	12	11	11	10	10	10	11	10	10	11	10	11
TGN	0	5	11	8	8	7	4	5	4	11	4	5

## Data Availability

The data presented in this study are available on request from the corresponding author.

## Supplementary Materials

The following are available online at https://www.mdpi.com/2076-2615/11/1/187/s1, Figure S1. Box plots for all real size measurements for each clade of Hynobius from the Republic of Korea. Each individual was measured three times and values averaged. The purpose of the dataset was to test for variations in morphology between clades, and in relation to the impact of patry and island effect. Figure S2. Box plots for all real size measurements of Hynobius from the Republic of Korea divided by SVL to remove bias due to size for each clade. Each individual was measured three times and values were averaged. The purpose of the dataset was to test for variations in morphology between clades, and in relation to the impacts of patry and island effects. Figure S3. Paratypes for all Hynobius species described in this paper: H. notialis sp. nov., H. geojeensis sp. nov. and H. perplicatus sp. nov. The field ID is given above each individual. Details on measurements are given on Table 9.
