# Supplementary material for: Disentangling the Impacts of Speciation, Sympatry and the Island Effect on the Morphology of Seven Hynobius sp. Salamanders
Source: Animals (Basel). 2021 Jan 14;11(1):187. doi: 10.3390/ani11010187 (PMC7830202; doi:10.3390/ani11010187)
Supplement: Supplementary file 1 [file animals-11-00187-s001.pdf]

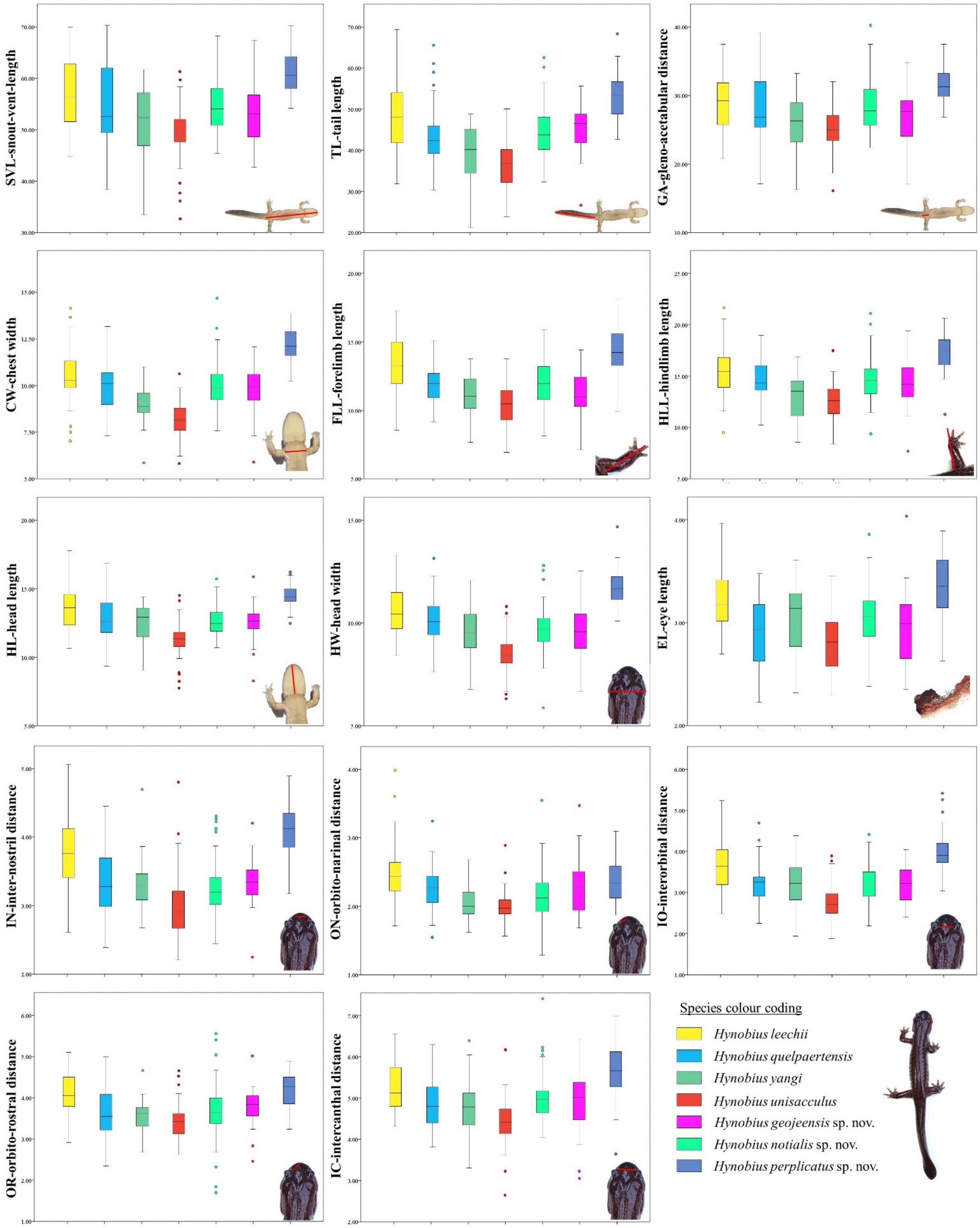

Figure S1. Box plots for all real size measurements for each clade of *Hynobius* from the Republic of Korea. Each individual was measured three times and values averaged. The purpose of the dataset was to test for variations in morphology between clades, and in relation to the impact of patry and island effect.

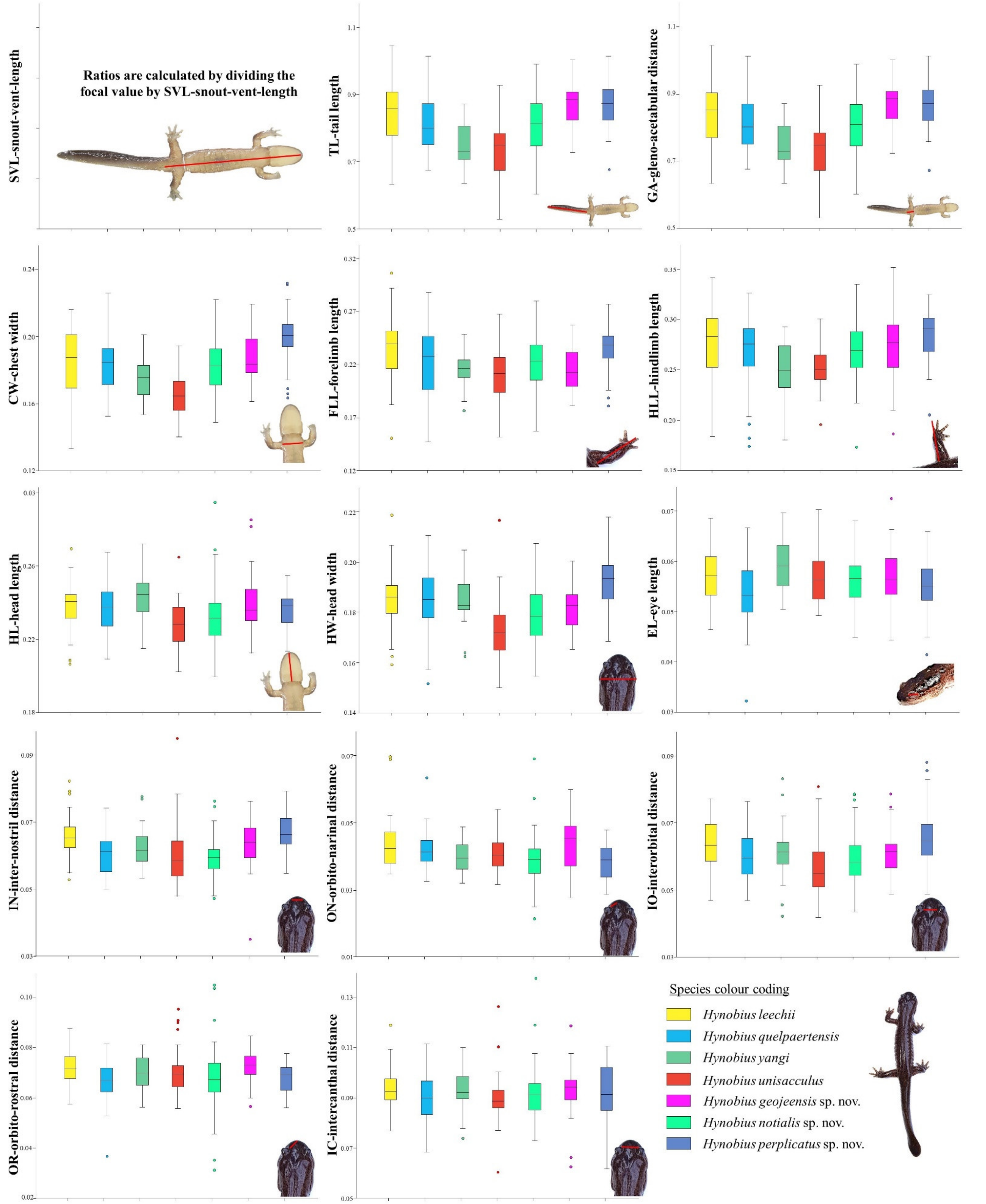

Figure S2. Box plots for all real size measurements of *Hynobius* from the Republic of Korea divided by SVL to remove bias due to size for each clade. Each individual was measured three times and values were averaged. The purpose of the dataset was to test for variations in morphology between clades, and in relation to the impacts of patry and island effects.

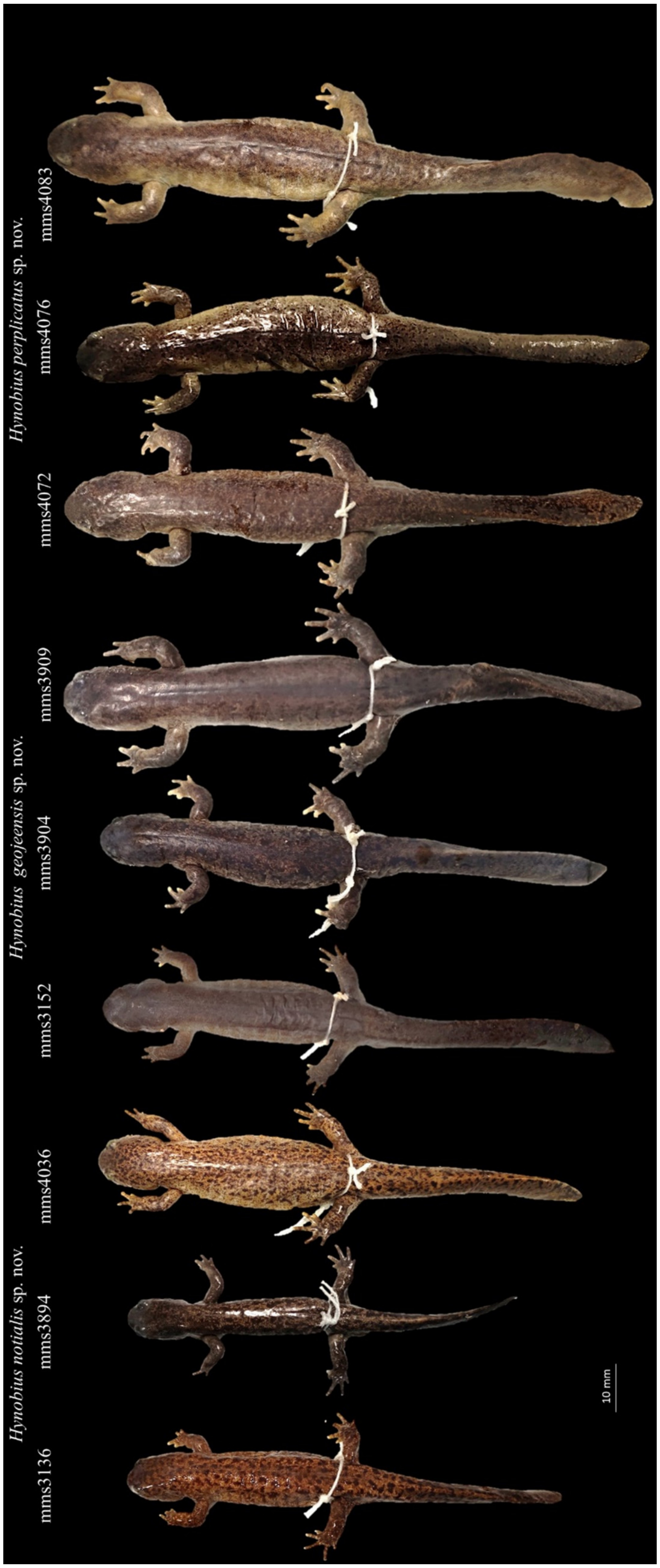

Figure S3. Paratypes for all *Hynobius* species described in this paper: *H. notialis* sp. nov., *H. geojeensis* sp. nov. and *H. perplicatus* sp. nov. The field ID is given above each individual. Details on measurements are given on Table 9.
